# Data mining reveals tissue-specific expression and host lineage-associated forms of Apis mellifera filamentous virus

**DOI:** 10.7717/peerj.16455

**Published:** 2023-11-14

**Authors:** Robert S. Cornman

**Affiliations:** U.S. Geological Survey, Fort Collins, Colorado, United States

**Keywords:** *Apis mellifera*, Apis mellifera filamentous virus, Kmer analysis, DNA viruses, Host-pathogen coevolution

## Abstract

**Background:**

Apis mellifera filamentous virus (AmFV) is a large double-stranded DNA virus of uncertain phylogenetic position that infects honey bees (*Apis mellifera*). Little is known about AmFV evolution or molecular aspects of infection. Accurate annotation of open-reading frames (ORFs) is challenged by weak homology to other known viruses. This study was undertaken to evaluate ORFs (including coding-frame conservation, codon bias, and purifying selection), quantify genetic variation within AmFV, identify host characteristics that covary with infection rate, and examine viral expression patterns in different tissues.

**Methods:**

Short-read data were accessed from the Sequence Read Archive (SRA) of the National Center for Biotechnology Information (NCBI). Sequence reads were downloaded from accessions meeting search criteria and scanned for kmers representative of AmFV genomic sequence. Samples with kmer counts above specified thresholds were downloaded in full for mapping to reference sequences and *de novo* assembly.

**Results:**

At least three distinct evolutionary lineages of AmFV exist. Clade 1 predominates in Europe but in the Americas and Africa it is replaced by the other clades as infection level increases in hosts. Only clade 3 was found at high relative abundance in hosts with African ancestry, whereas all clades achieved high relative abundance in bees of non-African ancestry. In Europe and Africa, clade 2 was generally detected only in low-level infections but was locally dominant in some North American samples. The geographic distribution of clade 3 was consistent with an introduction to the Americas with ‘Africanized’ honey bees in the 1950s. Localized genomic regions of very high nucleotide divergence in individual isolates suggest recombination with additional, as-yet unidentified AmFV lineages. A set of 155 high-confidence ORFs was annotated based on evolutionary conservation in six AmFV genome sequences representative of the three clades. Pairwise protein-level identity averaged 94.6% across ORFs (range 77.1–100%), which generally exhibited low evolutionary rates and moderate to strong codon bias. However, no robust example of positive diversifying selection on coding sequence was found in these alignments. Most of the genome was detected in RNA short-read alignments. Transcriptome assembly often yielded contigs in excess of 50 kb and containing ORFs in both orientations, and the termini of long transcripts were associated with tandem repeats. Lower levels of AmFV RNA were detected in brain tissue compared to abdominal tissue, and a distinct set of ORFs had minimal to no detectable expression in brain tissue. A scan of DNA accessions from the parasitic mite *Varroa destructor* was inconclusive with respect to replication in that species.

**Discussion:**

Collectively, these results expand our understanding of this enigmatic virus, revealing transcriptional complexity and co-evolutionary associations with host lineage.

## Introduction

Honey bee viruses are a major research topic in basic and applied entomology because of their persistent economic impact on apiculture (Jacques et al., 2017; Bruckner et al., 2023) and the complex, synergistic effects they exhibit with other stressors that challenge colony health (van Engelsdorp et al., 2010; Cornman et al., 2012; Goulson et al., 2015). Several RNA viruses are significant contributors to colony loss, particularly when multiple stressors are present (*e.g*., Dainat et al., 2012; Hou et al., 2014; Zheng et al., 2015; reviewed in Grozinger & Flenniken, 2019), and have been a focus of numerous surveys of pathogen prevalence, interaction, and host response. Functional genomic variation is increasingly studied (*e.g*. Ryabov et al., 2014, 2019), providing insight into viral ecology and mechanisms of colony loss.

In contrast, much less is known about DNA viruses in honey bees as the few known examples are not economically significant pathogens (Bailey, Carpenter & Woods, 1981; Grozinger & Flenniken, 2019). Among these is Apis mellifera filamentous virus (AmFV), a large double-strand DNA virus approximately 500 kilobases (kb) in length with evolutionary affinity to baculoviruses, nudiviruses, and hytroviruses (Gauthier et al., 2015; Yang et al., 2022). AmFV was first recognized as a DNA virus of honey bees by Clark (1978) and further characterized by Bailey, Carpenter & Woods (1981), who visualized a folded-filament genome structure within rod-like virions. The virus is geographically widespread but rarely linked to disease (Bailey, Carpenter & Woods, 1981; Hartmann et al., 2015; Beaurepaire et al., 2020; Li et al., 2023), although high virion concentrations give hemolymph a distinctive ‘milky’ appearance (Bailey, Carpenter & Woods, 1981). AmFV has been detected in diverse tissues and is likely transmitted both vertically and horizontally (Gauthier et al., 2015). AmFV sequence is detectable in the Varroa mite (*Varroa destructor*) (Gauthier et al., 2015), a major pest of *A. mellifera*, but replication in that species remains to be demonstrated (as distinct from passive acquisition of virions from parasitized honey bees).

Although not evidently a pathogen of concern for bee management, AmFV remains intriguing given the wide range of host-virus relationships that have been revealed for large DNA viruses of insects, from highly pathogenic to mutualistic (Lu & Miller, 1996; Roossinck, 2011; Strand & Burke, 2013). Furthermore, natural and recombinant DNA viruses have been exploited to manipulate host species, primarily for biocontrol (Moscardi, 1999). Little is currently known about the evolutionary ecology of AmFV or the molecular mechanisms of infection and host response. The first AmFV genome draft was reported by Gauthier et al. (2015), with a second accession reported by Yang et al. (2022) that is approximately 98% identical at the nucleotide level. Gauthier et al. (2015) recovered PCR products suggesting that circular forms of AmFV occur, consistent with proposed taxonomic affinities, but it is unclear to what extent linear or subgenomic forms might also occur. Most of the coding potential of the genome has remained hypothetical due to a paucity of apparent homology with known proteins (Gauthier et al., 2015; Yang et al., 2022), making it challenging to annotate with confidence, a characteristic shared with many other large DNA viruses (Iyer et al., 2006; Wang, Bininda-Emonds & Jehle, 2012). Similarity to baculoviruses is evidenced primarily by open-reading frames (ORFs) homologous to baculovirus repeated ORF (BRO) proteins and *per os* infectivity factor (PIF) proteins. Yang et al. (2022) placed AmFV within the viral clade Naldaviricetes, which also includes Baculoviridae, but AmFV fell closer to families Nudiviridae and Hytrosaviridae by their approach. However, it is not clear that the PIF sequences analyzed by those authors are actually orthologous across these clades, and Gauthier et al. (2015) recovered inconsistent phylogenetic relationships with various other genes that are generally more conserved than PIFs (*e.g*., thymidylate synthase, ribonucleotide reductase, and DNA polymerase B) and also more likely obtained by shared ancestry rather than horizontally (Iyer et al., 2006). As with coding content and phylogenetic position, little is also known about AmFV replication and gene expression, such as whether ORFs are expressed similarly in all honey bee tissues and whether ORFs are expressed in discrete stages during replication.

The present study was undertaken to search for evolutionary evidence of ORF validity (such as codon bias and purifying selection on coding sequence), identify host characteristics that covary with AmFV detection, and investigate tissue-specific variation in expression pattern. This was done by scanning accessions of the Sequence Read Archive (SRA) of the National Center for Biotechnology Information (NCBI), a primary repository of public high-throughput sequencing data. Sequence reads were subsampled from each accession meeting search criteria and exact sequence words of fixed length (“kmers”) that are representative of the AmFV genome were counted, followed by more computationally intensive analyses when AmFV was detected above a threshold. Kmer counting is a generic approach to metagenomic surveillance (Maillet et al., 2012) that has previously been applied to the Deformed wing virus complex (Cornman, 2017) and the Lake Sinai virus complex (Cornman, 2019) of honey bees.

## Methods

### SRA selection

SRA accessions were identified using the taxonomic filter for *A. mellifera* (taxid 7460, which includes subspecific taxa), accessed 08/25/22. DNA accessions were filtered to include only whole-genome shotgun (WGS) sequencing from a single platform (Illumina), with a minimum of five million reads and a minimum read length of 50 bases. RNA accessions were also restricted to the Illumina sequencing platform and with a specified sequencing strategy of “RNA-Seq”. RNA accessions were required to have at least 10 million spots (to facilitate transcriptome assembly and read mapping for positive accessions) and a minimum read length of 50. SRA accessions and metadata are tabulated in File S1. These metadata are also available as a U.S. Geological Survey data release (Cornman, 2023).

As BioSample metadata categories are largely user defined and vary greatly in vocabulary and specificity, I examined numerous user-submitted fields that were potentially informative as to tissue, caste, and developmental stage of samples as well as their geographic origin. Based on this information, I binned accessions into the following inferred categories: sex (male, female, or NA), developmental stage (larval/pupal, adult, or NA), and tissue (abdomen, head, thorax, whole body, or NA). Potential queen samples were identified by searching all BioSample metadata for the term “queen” (case insensitive) and reviewing matches manually to ensure they referred to the sequenced organism rather than an extraneous term such as “queen-right” or “black queen cell virus”. Queen samples were excluded from analysis, due to their small total number and the strong biological differences between queens and workers. Pseudoqueens or worker-derived queens were not considered queens for this purpose.

### AmFV kmer-picking and genome reconstruction

Kmers of size 31 were first extracted from AmFV accession NC_027925.1 using dsk v.2.3.0 (Rizk, Lavenier & Chikhi, 2013) and then subsampled to a total of 3,000 single-occurrence kmers without matches in the *A. mellifera* genome or transcriptome (Amel_HAv3.1, GCF_003254395.2 (Wallberg et al., 2019)). NC_027925.1 was used as the primary reference genome instead of a more recent accession (OK392616.1) because it has fewer ambiguous characters (0.04% *vs* 1.01%) and the latter appears truncated by approximately 3 kb relative to other AmFV genome sequences generated by this study (see Results). The two accessions are otherwise very similar, with a nucleotide identity of 98.874% at non-ambiguous, aligned positions.

*A. mellifera*-specific kmers were also counted so that the relative abundance of AmFV to host sequence could be estimated. These host-specific kmers were obtained by extending the coding-sequence specific 25-mers used in Cornman (2017) an additional six bases. To estimate the degree of African ancestry (Wallberg et al., 2014) in host genomic sequence, two kmers were extracted from the 18S rRNA gene that differentiate European *A. mellifera* ancestry from African ancestry (“A-lineage” hereafter). This strategy was validated in two ways: (1) by comparing the relative abundance of these two kmers with kmer pairs representing previously identified single nucleotide polymorphisms (SNPs) differentiating the two lineages (Chapman et al., 2015), and (2) by comparing 18S frequencies in geolocated samples from Africa and from known hybrid zones between African and European lineages of *A. mellifera* (Fig. S1). Only the two alternative 18S kmers were used to calculate A-lineage proportions because the 18S locus has better sampling properties than single-copy SNPs: there are 85 annotated copies of the rRNA locus throughout assembly HAv3.1 (Wallberg et al., 2019) and within-lineage variation is expected to be low due to purifying selection (Rooney & Ward, 2005) and concerted evolution (Eickbush & Eickbush, 2007). In contrast, single-copy nuclear SNPs are sampled with higher stochasticity and are more likely to have adjacent variation that would bias kmer counts.

DNA accessions with high numbers of kmers, operationally defined as at least 5,000 total AmFV kmer counts and a ratio of AmFV to *A. mellifera* kmer counts greater than one, were downloaded in full and mapped to the reference to generate consensus sequences for phylogenetic analyses. Read mapping was performed with bowtie2 v.2.4.5 (Langmead & Salzberg, 2012) with “fast” and “end-to-end” pre-set parameter switches. Alignments were then sorted and filtered at a Phred-scaled mapping quality of 30 with samtools v. 1.12 (Li et al., 2009). Consensus sequences were generated with bcftools v. 1.10.2 (Danecek et al., 2021) using the commands mpileup (with the map-quality adjustment set to 50), call (using the consensus biallelic model and with ploidy set to haploid), and consensus. Positions with less than 3X coverage were converted to N in the final consensus sequence. Whole-genome phylogenies drawn with these initial consensus sequences revealed three major clades of AmFV as well as a pattern of reduced mapping rates in samples that fell within the two novel clades. This pattern implies that AmFV sequences from the novel clades were too divergent from the original reference genome for read mapping to reliably reconstruct genomic variation. I therefore used Spades v. 3.15.2 (Bankevich et al., 2012) to perform *de novo* assembly (with assembly mode set to “isolate”) and scaffolded the resulting contigs with ragtag v. 2.1.0 (Alonge et al., 2022) using NC_027925.1 as the scaffolding reference (the default scaffold gap was 100 bp). For the two newly identified clades, the least-gapped assembly exceeding 490 kb in length that was recovered by this strategy was used as a new reference for read mapping within each novel clade as well as for kmer picking. The revised kmer-picking strategy identified a set of 3,000 kmers shared among the three reference genomes and 3,000 kmers unique to each (for a total of 12,000). These kmer classifications are necessarily approximate at present, as there is insufficient information to verify whether “clade-specific” kmers reflect genuine fixed differences.

### Kmer searching

Kmer searching was performed on the YETI high-performance computer system of the U.S. Geological Survey (https://www.usgs.gov/core-science-systems/sas/arc/usgs-yeti-supercomputer). Five million reads for DNA accessions and one million reads for RNA accessions were accessed randomly, without regard to orientation in a read pair, using the fasterq-dump utility of sratoolkit v.3.0.0 (https://github.com/ncbi/sra-tools). Fewer RNA reads were searched because transcriptome complexity is lower than genome complexity in large eukaryotic genomes. Singleton kmers were ignored in a given sample as these are usually enriched in sequencing error and greatly increase the total search space (kmer counting workflows often include abundance filtering and dsk excludes singleton kmers by default (Rizk, Lavenier & Chikhi, 2013)). All accessions were scanned for all kmer lists (AmFV shared, AmFV clade-specific, *A. mellifera* coding sequence, and *A. mellifera* 18S), although 18S kmers were not tabulated for RNA accessions because rRNA is intentionally depleted during RNA-Seq library preparation. Kmer lists extracted from each reference are provided in File S2. Shared AmFV kmers were distributed across the genome and usually recovered at similar relative abundances with low coefficients of variation (Fig. S2), indicating good representativeness. Two 10-kb genomic windows that had higher variance coincided with strong, local shifts in nucleotide composition (see Results), which is known to affect library capture (Dohm et al., 2008).

DNA accessions with zero AmFV kmer matches were considered negative for AmFV, whereas accessions with at least ten AmFV kmer counts were considered positive. Accession with 1–9 total counts were censored, that is they were considered not definitively in either category, since low numbers of AmFV kmers could potentially be cross-talk contaminants from other multiplexed samples (Edgar, 2018). These thresholds are subjective and imperfect, as it remains unclear how to best model and mitigate for cross-talk (Edgar, 2018), particularly in the absence of control samples.

### Consensus genome sequence generation

DNA accessions with high levels of AmFV as defined above were downloaded in full for mapping to clade-specific reference genomes for within-clade population genetic statistics, provided that a single AmFV clade was dominant (see Results), *i.e*. constituted 80% or more of total AmFV kmers. Consensus sequences were generated as described above. RNA accessions with 1,000 or more total kmer counts and a relative abundance of at least 0.01 AmFV kmers per *A. mellifera* kmer were downloaded in full for transcriptome assembly and read mapping. Transcriptome assembly was performed with Spades with minimum coverage set to 3 and automatic error correction disabled. To identify long AmFV transcripts, assembled contigs were searched against predicted AmFV proteins using default BLASTX parameters, and the longest identified contig from each sample was aligned to reference NC_027925.1 using MAFFT v7.480 (Katoh et al., 2002).

A phylogeny of all AmFV genome consensus sequences was generated with MEGA X (Kumar et al., 2018) using neighbor joining, the maximum composite likelihood mutational model, and the default gamma model of rate variation (five rate categories with shape parameter set to one). Bootstrap values were calculated from 1,000 resampled data sets. To test alignment-free phylogenetic comparisons using kmer composition profiles only, pairwise distances were computed between samples with at least 1,000 unique kmers counted using the triangle command in Mash v. 2.3 (Ondov et al., 2016). Triangle distance matrices were imported into MEGA X to generate neighbor-joining trees of SRA accessions.

### ORF evolutionary analysis

A six-genome alignment was generated with MAFFT from four *de novo* draft genomes (two each from the novel clades described above) and two reference genome accessions (NC_027925.1 and OK392616.1) (File S3). ORF alignments were then cut from the multi-genome alignment by padding the annotated coordinates of ORFs for NC_027925.1 based on the number of preceding gaps in the six-genome alignment. The expected coding sequence extracted from NC_027925.1 with gffread (Pertea & Pertea, 2020) was appended to each ORF alignment temporarily during this process to confirm preservation of coding frame, which was manually reviewed in BioEdit v. 5.0.9 (Hall, 1999). ORF alignments are provided in File S4. Signal peptides were identified using SignalP 6.0 (Teufel et al., 2022).

The ENC’ measure of codon bias (Novembre, 2002) was estimated for ORF alignments with the R package coRdon v.1.0.3 (Elek, Kuzman & Vlahovicek, 2023). The ratio of nonsynonymous to synonymous substitutions in each alignment (ω) was estimated with both the BUSTED program of the HYPHY package v. 2.5.32 (Smith et al., 2015) and PAML v. 4.9j (Yang, 2007). Likelihoods were estimated in triplicate (to evaluate convergence) and averaged for PAML models 1 (two ω classes without positive selection) and 2 (three ω classes including positive selection). The six-genome guide tree was generated in MEGA X using the neighbor-joining algorithm described above. ORF alignments were manually trimmed of poorly aligned or repetitive low-complexity regions, as codon-level orthology cannot be reliably determined for such regions. For all six-sequence ORF alignments, the ω was estimated using model 1 of PAML, however only alignments of at least 150 codons after trimming were analyzed for positive selection due to the challenge of detecting excess nonsynonymous substitutions in short alignments with relatively few substitutions. Raw *P*-values were adjusted for false discovery using the Benjamini-Hochberg method of the R function p.adjust (R Core Team, 2013).

### Statistical analysis

The pairwise number of differences between reference genomes was estimated in sliding windows using DnaSP v 6.0 (Rozas et al., 2017). Tajima’s D and nucleotide diversity (π) were calculated in sliding windows of genomic sequence with vcftools v. 0.1.17 (Danecek et al., 2011) using the haploid-compatible version available from https://github.com/vcftools/vcftools/pull/69. PAST3 (Hammer, Harper & Ryan, 2001) was used to compute contingency tests and perform Kruskal-Wallis tests of relative AmFV abundance. Pairwise similarities and alignment consistency scores were calculated with T-Coffee v. 13.45.0 (Notredame, Higgins & Heringa, 2000). Tandem repeats were identified with the Tandem Repeats Finder server (Benson, 1999) available at https://tandem.bu.edu/trf/trf.html, specifying a minimum score of 100 and a period size of 8 to 20. Insect promoter motif scores were generated with the Neural Network Promoter Prediction tool (Reese, 2001) available at https://fruitfly.org/seq_tools/promoter.html.

ORF-level read counts in RNA alignments were tabulated with the bedcov function of samtools. Among-sample Spearman correlations of ORF-level read counts were plotted as heatmaps with the R package corrplot v. 0.92 (Wei & Simko, 2013). Among-sample and among-ORF expression clusters were generated and visualized using clustergrammar (Fernandez et al., 2017) with correlation as the specified method. To account for the nonindependence of NGS count data (Gloor et al., 2017), raw ORF read counts were first transformed using a log-ratio approach prior to estimating correlation coefficients and expression clustering. Specifically, counts were converted to proportions within each accession, divided by the geometric mean of all such proportions in the sample (zeroes excluded), and the base ten logarithm taken. Since this operation results in both positive and negative values, a common scalar was then added to all non-zero values such that the minimum non-zero value was 0.01. This procedure allows correlational analysis of compositional data while ensuring that zero values are at the bottom of the distribution rather than intermediate.

## Results and discussion

### Structure and phylogeny of reconstructed genome sequences

**Figure 1:**
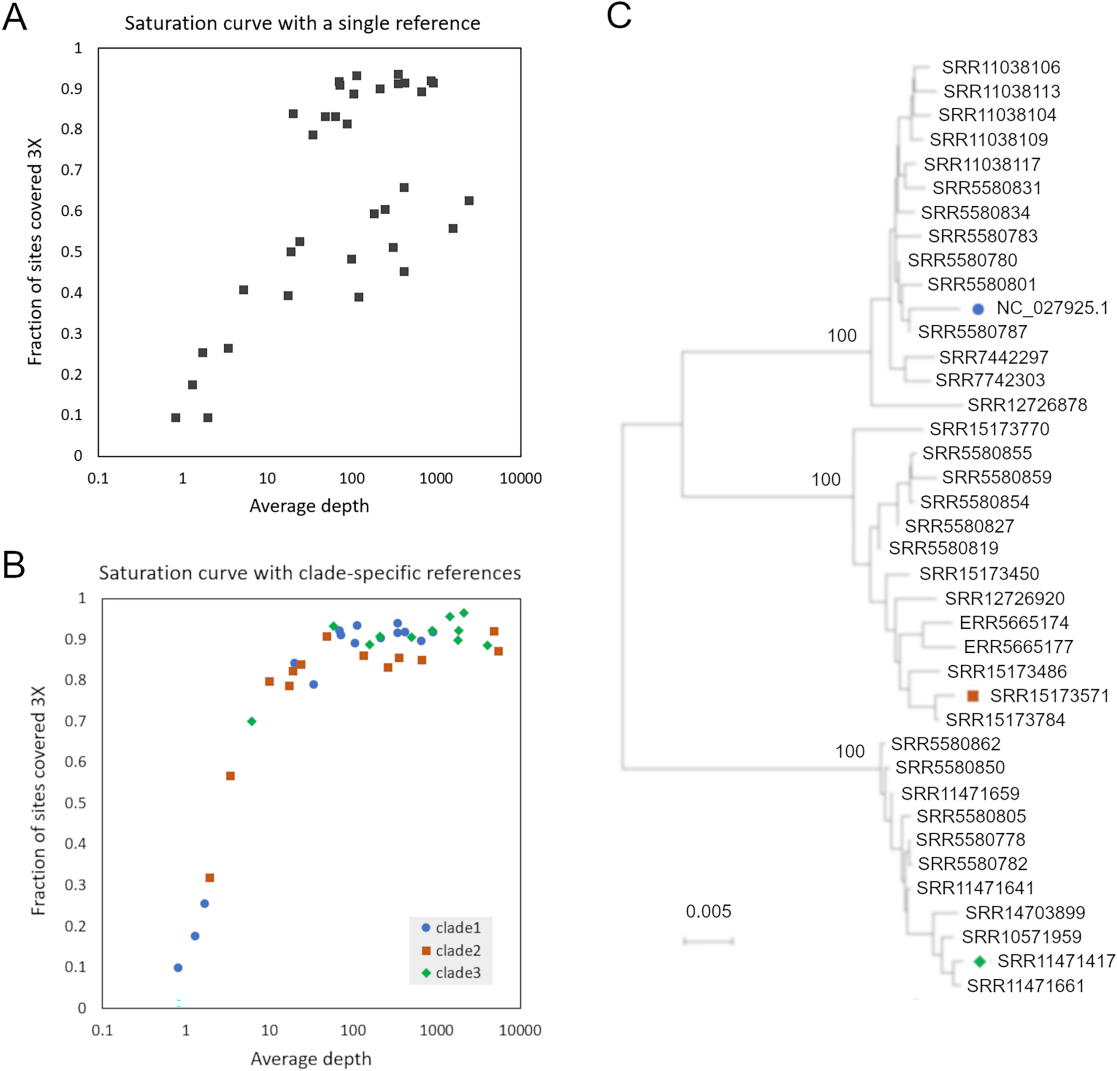
Read mapping and phylogenetic analysis indicates at least three distinct clades of Apis mellifera filamentous virus (AmFV). (A) Proportion of genome positions with at least 3X coverage as a function of genome-average read depth, for accessions mapped to reference NC_027925.1. (B) Proportion of genome positions with at least 3X coverage as a function of genome-average read depth, for accessions mapped to one of three clade-specific references indicated by the legend. (C) Consensus-sequence phylogeny of all accessions with a single dominant clade of AmFV, as well as the reference genomes used for mapping.

SRA accessions were initially searched for a set of 31-mers randomly extracted from AmFV reference genome NC_027925.1 (and not found in *A. mellifera* reference genome HAv3.1). However, when reads from accessions with high kmer counts were mapped to that reference genome, per-base coverage failed to converge toward 100% as the number of mapped reads increased (Fig. 1), a phenomenon most pronounced for hosts with A-lineage ancestry. A phylogenetic analysis of these initial consensus genome sequences indicated distinct clades were present (Fig. 1B). These observations implied that the AmFV genomes represented in those SRA accessions were too divergent for read mapping to a single reference sequence to adequately represent natural variation.

To improve the reconstruction of these more divergent AmFV sources, I performed *de novo* metagenomic assembly and used reference-guided scaffolding in an attempt to recover draft reference genomes for the divergent clades. Four SRA accessions (SRR11471661, SRR11471417, SRR15173571, and SRR558059) yielded AmFV assemblies ranging from 493,854–566,180 bp in length and with gap proportions ranging from 0.3-7.1%. These four genome sequences were aligned with reference accessions NC_027925.1 and OK392616.1 for evolutionary analysis (File S3). The six-genome phylogenetic topology was consistent with the initial genome phylogeny derived from a single reference and again supported three major clades of AmFV (Fig. S3). Using a distinct reference genome for each of the three clades yielded coverage curves that approached saturation for all mapped samples (Fig. 1C). This three-clade consensus strategy is corroborated by an alignment-free phylogenetic approach using the kmer distance computed by Mash (Fig. S3). Aligned consensus genomes used in the phylogenetic analysis are provided in File S5.

### Comparative genome structure

**Figure 2:**
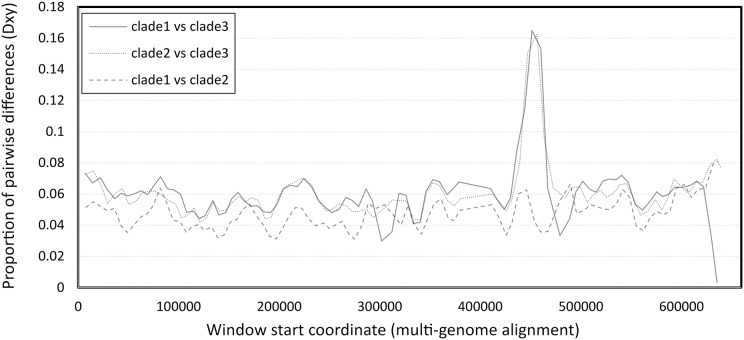
Pairwise divergence among the three clades of Apis mellifera filamentous virus (AmFV). Divergence was calculated in consecutive windows of 10,000 aligned positions.

The number of pairwise differences (Dxy) was similar across much of the genome, except for a strong peak of divergence between clade 3 and the other two clades in the vicinity of 450–460 kb of the six-genome alignment (Fig. 2). This approximately corresponds to the 340–350 kb region of the reference clade 1 genomes (NC_027925.1 and OK392616.1). Localized genomic regions of high divergence in both coding and noncoding sequence, without loss of ORF integrity, could indicate recombination rather than diversifying selection or neutral evolution; further evidence supporting this hypothesis is discussed below.

**Figure 3:**
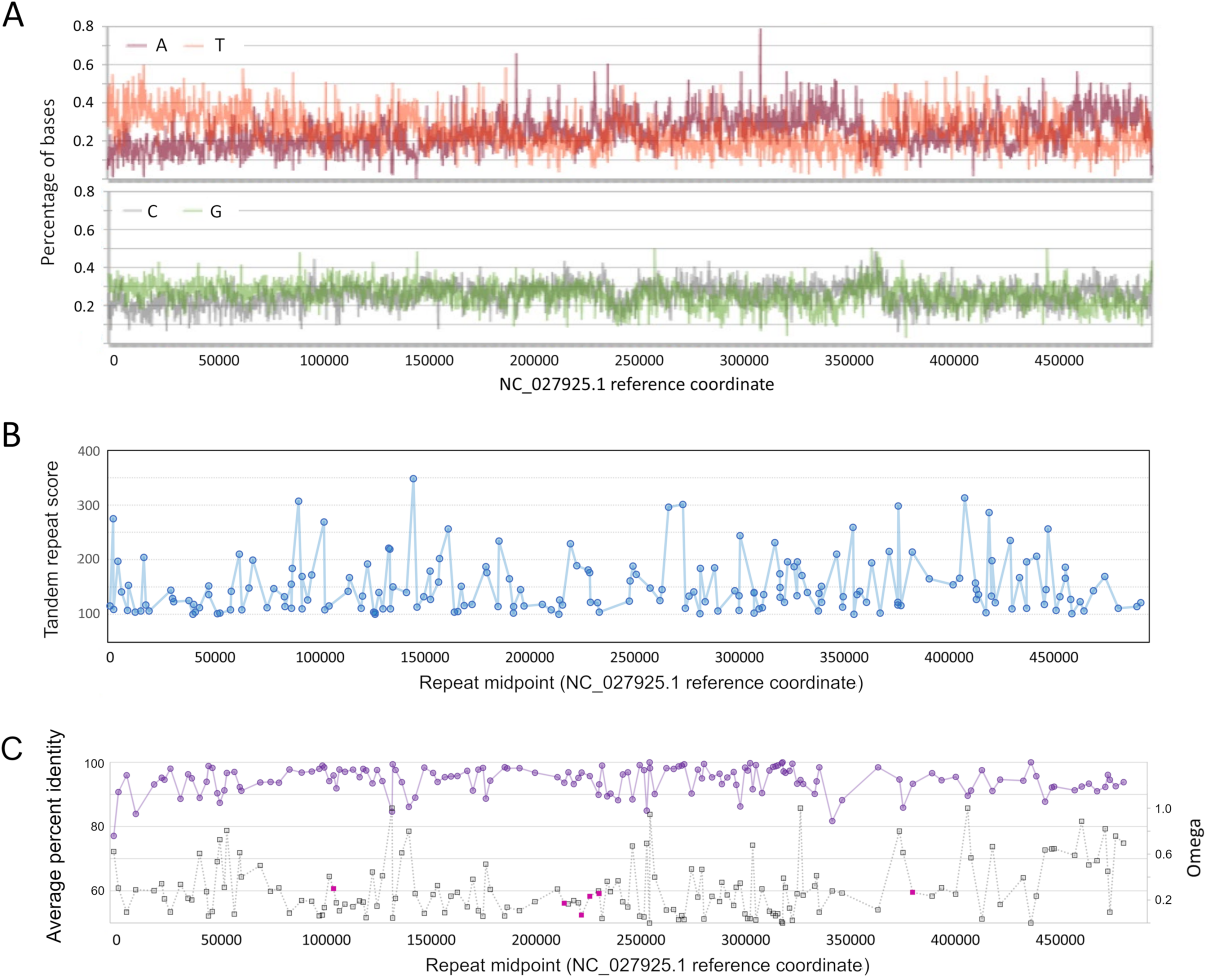
Genomic characteristics of Apis mellifera filamentous virus (AmFV). (A) Nucleotide composition on the plus strand of AmFV. (B) Tandem repeat scores for all 8–20 nucleotide motifs in the AmFV genome scoring above 100 (see text for details). (C) Pairwise protein identity of predicted proteins in six-genome alignments (purple line) and evolutionary rates (gray line) for the corresponding nucleotide sequences. ORFs encoding potential signal peptides are shaded magenta. Lines in panels (B) and (C) are added to illustrate trends.

That nucleotide composition varies along the AmFV genome has been previously reported (Gauthier et al., 2015), but it can be further shown that composition biases are pronounced at the strand level as well, oscillating between strong A and T bias (Fig. 3A) on the plus strand. Homopolymer runs of A or T appear to contribute to these biases (Fig. S4); for example, there is a strong shift in strand bias *circa* 300 kb towards increasing T on the plus strand, which corresponds to a large increase in the number of T homopolymers of length five or more. Strand-specific nucleotide biases have been shown to be associated with replication origins and strand-specific transcription in double-stranded DNA virsues, and skew is typically greater for AT than GC (Francino & Ochman, 1997; Grigoriev, 1999). However, the observation that homopolymer runs contribute to the pattern suggests that the biases may have functional significance rather than merely reflecting polymerase activity. For example, Grigoriev (1999) proposed that variation in nucleotide availability during the cell cycle could favor isochore evolution in asymmetrically replicating dsDNA viruses.

As found for related DNA viruses such as baculoviruses and nudiviruses (Wang, Bininda-Emonds & Jehle, 2012; Williams, Bergoin & van Oers, 2017), short tandem repeats of 8–20 bp repeat units are also distributed throughout the AmFV genome, although repeated motifs are not necessarily compositionally biased (Fig. 3B). A total of 187 repeats with a score of 100 or more (File S6) were identified by Tandem Repeats Finder (Benson, 1999).

### Evolutionary assessment of ORF annotations

I manually classified ORFs annotated for NC_027925.1 as well-conserved, unconserved, or indeterminate in the six-genome alignment. I did not consider missing start or stop codon positions as disqualifying provided that at least 100 aligned codons were found, as such differences may represent errors in the original annotation, sequencing error, or genuine evolutionary divergence. Of 241 hypothetical ORFs annotated for NC_027925.1, I classified 155 as well-conserved, 52 as unconserved, and 34 as undetermined due to missing data or extensive low-complexity sequence. In comparison, Yang et al. (2022) annotated 197 ORFs for OK392616.1. ORF annotations and properties are summarized in File S7 and follow the existing naming convention for NC_027925.1 (*i.e*., “gp” followed by a three digit number representing consecutive order along the reference sequence).

**Figure 4:**
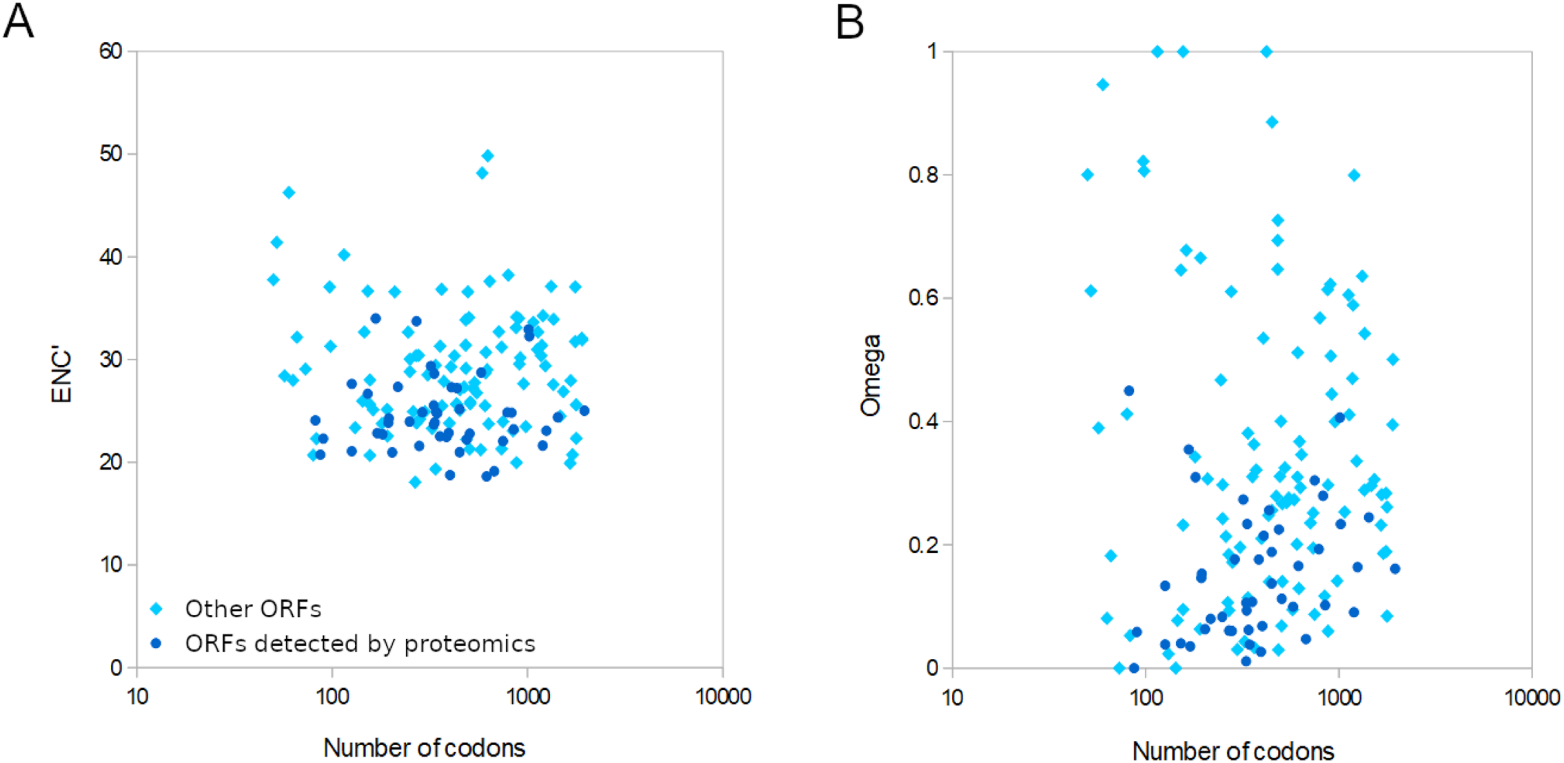
Relative conservation of codons in open reading frames (ORFs) of Apis mellifera filamentous virus (AmFV). ORFs detected by proteomics in a published study (see text for details) are contrasted with other annotated ORFs. (A) Effective number of codons (ENC’) of each ORF relative to bee ribosomal proteins, which are presumed to be highly expressed by the host. ENC’ is plotted relative to ORF length to demonstrate no consistent effect on codon bias estimates. (B) Ratios of nonsynonymous to synonymous substitutions (evolutionary rates) of each ORF, again plotted relative to ORF length since that influences the power to estimate such rates.

For the 155 well-conserved alignments, 124 had an estimated ω < 0.5 and 141 had an ENC’ less than 35 relative to honey bee ribosomal proteins (Figs. 4 and S7). Strongly biased codon usage results in an ENC’ much less than the maximum value of 61 codons and is generally interpreted as resulting from selection for high expression (Novembre, 2002). ORFs meeting both criteria numbered 119, including all ORFs identified by proteomics by Yang et al. (2022). For comparison, the majority of honey bee coding sequences had ENC’ values above 40 (Fig. S5), indicating less biased codon usage. As would be expected for any eukaryote, protein evolutionary rates in genus *Apis* are much more constrained: 95.8% of *Apis* genes analyzed by Fouks et al. (2021) had ω estimates less than 0.5 (File S2 of that reference).

No strand bias was evident among conserved ORFs: 75 occur on the plus strand, 78 on the minus strand. However, the genome 3′ of the compositionally biased region noted above at ~360 kb (Fig. 3C) has a lower density of ORFs and these are less conserved on average. Genomic regions immediately upstream of ORFs are not enriched in insect core promoter candidates: 25/155 (16.1%) ORFs had candidate promoters scored above the default threshold score of 0.8, whereas 224/1,000 (22.4%) randomly selected 300 bp genomic regions also scored this high.

Several ORFs with putative signal peptides were identified, which can have key roles in viral replication (*e.g*., Okamoto et al., 2008). ORFs with ‘covered’ signal peptides downstream of the annotated start codon were also identified, which merit attention because this sequence context has been shown to be functionally important for virulence in a baculovirus (Hao et al., 2022). Most ORFs with candidate signal peptides were clustered in the genome (gp96, gp100, gp101, and gp103; Fig. 3C). One of these, gp103, had a high number of inferred substitutions (“tree length” in File S7) and yielded low *P*-values for positive diversifying selection by both analysis methods (*P* = 2.49E−5 with BUSTED and *p* = 0.0035 with PAML). However, FDR-adjusted *P*-values were not both below 0.05, the conservative criterion chosen for inferring positive selection given the relatively low codon number and short branch lengths for these alignments. The potential for positive selection on gp103 should be revisited as more data becomes available.

For functional annotation of distant homology, it is often preferable to weight known protein domains, for example by using domain-enhanced (DELTA) BLAST (Boratyn et al., 2012). Of the 155 ORFs classified as conserved, 37 (23.8%) contained at least one domain match to NCBI’s conserved domain database (CDD; Marchler-Bauer et al., 2005) at an expectation of 1E−10 or lower (File S7). The detected domain homologies help clarify ORFs with potential roles in viral replication, virulence, and metabolism. The only AmFV ORF previously annotated with an unambiguous role in genome replication is gp074, which contains DNA polymerase B domains (Gauthier et al., 2015; Yang et al., 2022). Other ORFs with hypothesized roles in replication are gp042 and gp095, which have previously noted similarities to integrase and ligase proteins (Gauthier et al., 2015, Yang et al., 2022). Other expected functional domains based on related viruses and general requirements for double-stranded DNA replication (Iyer et al., 2006; Wang, Bininda-Emonds & Jehle, 2012; Rampersad & Tennant, 2018), such as helicase and topoisomerase activities, have not been found or were supported by only weak homology. DELTA BLAST clarified that the closest domain-level homology of gp042 is to tyrosine site-specific recombinase, which are found in topoisomerases and site-specific recombinases as well as phage integrases. In the baculovirus *Autographa californica* multiple nucleopolyhedrovirus, a tyrosine recombinase is required to complete genome packaging within capsids (Vanarsdall, Okano & Rohrmann, 2006). ORF gp095 contains both a zf-PARP polyadenylation domain and a DNA ligase I domain. Proteins with zf-PARP domains act to remodel chromatin around DNA breaks and also bind the breaks (Lilyestrom et al., 2010; Ali et al., 2012), whereas DNA ligase I activity is required for lagging strand synthesis of double-stranded DNA (Timson, Singleton & Wigley, 2000). Additional domains detected by DELTA BLAST that might support replication include an endonuclease V domain (gp122) most similar to bacterial RecB, which has helicase activity (Dillingham, Spies & Kowalczykowski, 2003).

Viral packaging often involves the co-option of host cytoskeleton components (Khorramnejad et al., 2021), and several ORFs that are candidates for interactions with the cytoskeleton were found. These include gp011, which as previously recognized (Gauthier et al., 2015;, Yang et al., 2022) contains a kinesin molecular-motor domain, and gp237, which contains a PAT1 domain that can associate with kinesin; both of these protein families have been shown to be recruited by DNA viruses during virion assembly and export (Benboudjema et al., 2003; Pegg et al., 2021). The transcriptional machinery of AmFV remains undocumented, however DELTA-BLAST indicates that the gp152 protein may associate with an RNA polymerase or core transcription factor, as it contains an RNA cleavage domain found in all three eukaryotic RNA polymerases (Ruan et al., 2011). ORF gp103 may be a candidate for transcript processing, as it contains a ribonuclease E domain that is essential for selective processing of a suite of RNAs in bacteria (Bechhofer & Deutscher, 2019).

### Population-genetic variation within AmFV clades

Population-genetic statistics were computed for each clade using sliding-window analysis. One caveat of this analysis is that SNP ascertainment is potentially unequal among clades since the underlying sample types are heterogeneous in terms of the numbers of bees represented, their geographic provenance, and the relative abundance of AmFV. Nonetheless, the total number of accessions included for each clade was similar (*n* = 14 for clade 1, *n* = 11 each for clade 2 and clade 3) and since variants were called as consensus haploid genotypes, the influence of low-frequency variants within samples should be minimal. In fact, genomic patterns of nucleotide diversity were broadly similar across clades (Fig. S6), although π declines toward the 3′ end of the genome in clades 1 and 3. Tajima’s D in 10-kb genomic windows (coding status ignored) was near zero on average in all three clades, indicating no net difference in the distribution of rare alleles that would suggest strong demographic events or selection patterns in recent evolutionary history.

The high divergence of clade 3 from the other two clades in a limited genomic window (Fig. 2) could parsimoniously be explained by past recombination with an unknown, divergent AmFV-like virus. The possibility of recombination between the AmFV lineages described here and more divergent lineages is further supported by a pattern of extreme differentiation of the genome sequence reconstructed from accession SRR5580859. A region containing multiple ORFs at the 3′ of that genome is characterized by extreme pairwise divergence independent of coding status, or codon position within ORFs (Fig. S7A). While a definitive recombination breakpoint was not found upstream, a well-defined recombination breakpoint was found downstream of this region (Fig. S7B). ORFs within the region are highly divergent in sequence (Fig. S7C) but fully align with other genomes at the codon level, indicating that those coding sequence have not been pseudogenized.

### AmFV detection rates in DNA and RNA accessions

Of 2,411 WGS libraries with at least five million reads and an average read length of at least 50, AmFV kmers were detected above threshold in 396, whereas 189 were censored as ambiguous (having fewer than ten total AmFV counts). The detection rate was therefore 21.6% overall. The number of RNA SRA accessions scanned was 3,174 (File 1), of which 151 accessions had counts above threshold, 2,840 had zero counts, and 183 accessions were censored as ambiguous (1–9 counts). The overall detection rate for one million scanned RNA reads was therefore 5.0%.

The AmFV detection rate in DNA was *prima facie* higher in females (302/814 SRA accessions, or 37.1%) than males (84/1376 SRA accessions, or 6.1%). However, the total number of BioProjects represented by DNA accessions was relatively small (*n* = 42) and strongly skewed (number of accessions ranged from 1–817 per BioProject). An explicitly stratified analysis of detection rates was not practical given that few BioProjects include multiple categories of tissue, stage, or sex. I therefore tabulated DNA detection rates by metadata category within BioProjects with at least 30 accessions analyzed (Table 1), which revealed a systematic bias in male and female detection rates: males were typically analyzed as individuals whereas females were often aggregated in pools. When only BioProjects that sampled individuals were considered, males and females appeared to have similar AmFV detection rates, except in a few cases involving hosts of hybrid origin (Table 1, discussed further below).

**Table 1:** Apis mellifera filamentous virus (AmFV) detection rates in DNA accessions of the Sequence Read Archive (SRA), for BioProjects with at least thirty analyzed accessions. The description column lists whether sequenced samples were individuals or pools of individuals, the subspecies of *Apis mellifera* if known, and the geographic regions sampled, as inferred by the author from BioProject descriptions and BioSample metadata.

BioProject	Sex	Tissue	Negative	Positive	Rate	Description
PRJNA666033	Female	Head	12	25	0.676	Pools, multiple subspecies, Europe
PRJNA622776	Female	Thorax	58	105	0.644	Individuals, scutellata x mellifera hybrid zone, Argentina and California
PRJNA592197	Female	Larval/pupal	15	26	0.634	Individuals, capensis x scutellata hybrid cross, southern Africa
PRJNA605407	Female	Whole body	28	48	0.632	Pools, mellifera, US domestic stocks
PRJNA385500	Female	Whole body	39	36	0.480	Individuals, mostly feral mellifera, California (implicit hybrid zone)
PRJNA729035	Female	NA	19	12	0.387	Individuals, multiple subspecies across range
PRJNA729035	Female	Thorax	71	7	0.090	Individuals, multiple subspecies across range
PRJNA292680	Female	Whole body	31	2	0.061	Individuals, mellifera, USA
PRJNA357367	Female	Thorax	33	1	0.029	Individuals, scutellata, Kenya
PRJNA216922	Female	NA	36	0	0.000	Individuals, multiple subspecies
PRJNA311274	Female	Thorax	30	0	0.000	Individuals, mellifera, France
PRJNA507348	Female	Whole body	70	0	0.000	Individuals, capensis, southern Africa
PRJNA311274	Male	Thorax	723	64	0.081	Individuals, mellifera, France
PRJNA596071	Male	Thorax	55	4	0.068	Individuals, multiple subspecies, Europe
PRJNA252997	Male	NA	43	2	0.044	Individuals, mellifera, China
PRJNA381313	Male	Thorax	86	2	0.023	Individuals, mellifera, USA
PRJEB16533	Male	NA	113	2	0.017	Individuals, mellifera, Europe
PRJNA516678	Male	NA	171	0	0.000	Individuals, mellifera, Europe

### AmFV detection rates and clade abundance vary by host genetic background

**Figure 5:**
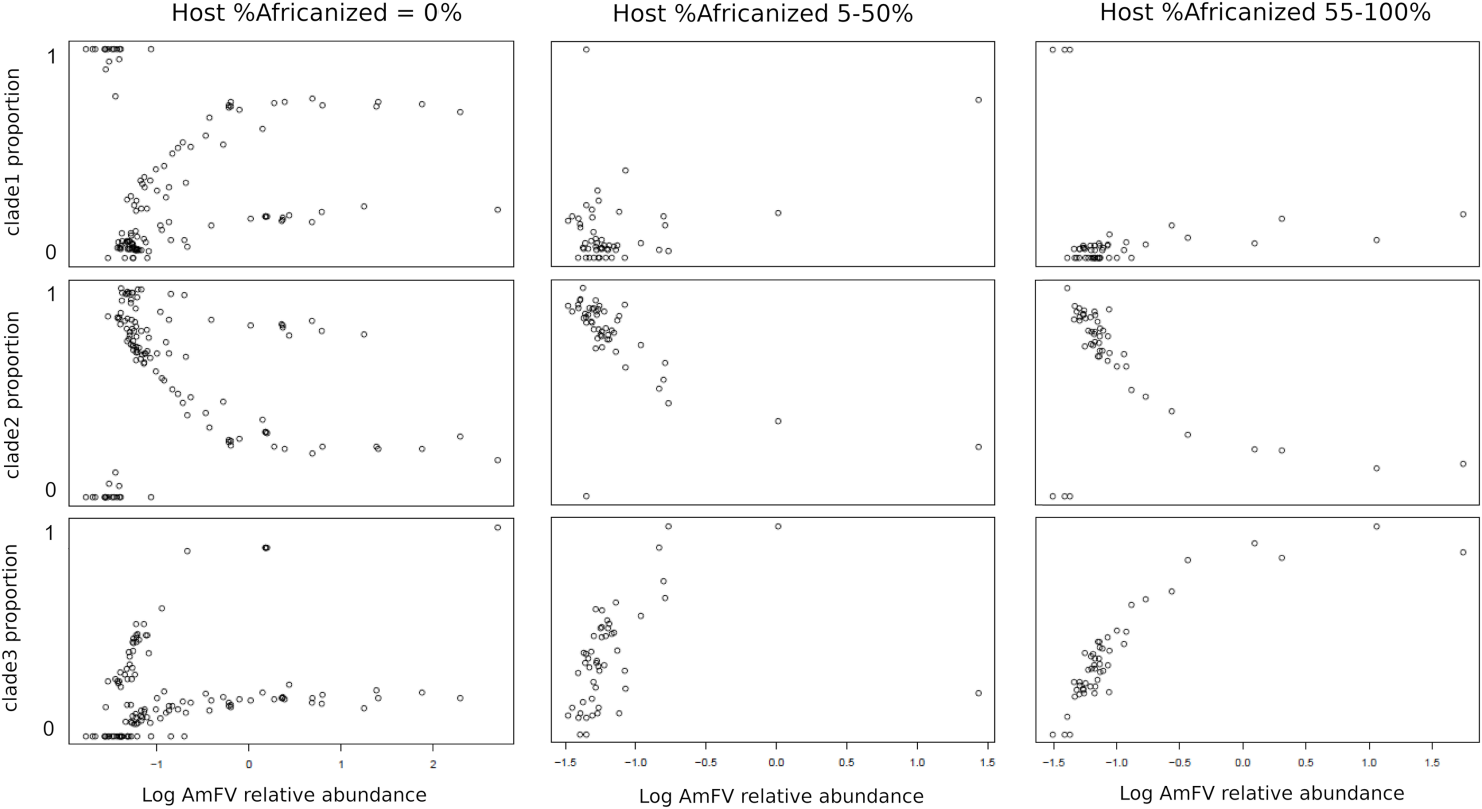
The proportion of Apis mellifera filamentous virus (AmFV) clades depends on host ancestry and overall AmFV relative abundance. Each column of panels represents a qualitative bin of A-lineage ancestry in the hosts, whereas each row represents the proportion of an AmFV clade. Points indicate the proportion of AmFV clades as a function of increasing overall abundance relative to the host. Note the horizontal axes are log-scaled and ranges differ between columns.

The relative abundance of AmFV clades in samples depended on the proportion of A-lineage ancestry of the hosts (Fig. 5). Since the ratio of A-lineage kmers is a continuous but unevenly distributed variable, I binned accessions into three categories reflecting zero, minority, or majority A-lineage ancestry, specifically, 0%, 5–50%, or 55–100% A-lineage kmers. The small gaps between bin ranges were introduced to mitigate stochastic variation near bin boundaries (*e.g*., very low but nonzero A-lineage ancestry or nearly equal numbers of A-lineage and non-A-lineage kmers), as well as to make the bins more qualitative in nature. The resulting plots indicate that clade 1 AmFV dominates when the host had zero A-lineage kmers and the relative abundance of AmFV overall is very low. However, as the relative abundance of AmFV increased in these hosts, infections were invariably mixtures of clades and these mixtures occurred at relatively discrete ratios, possibly indicating recombinant forms. In hosts with A-lineage ancestry, clade 2 was dominant at low relative abundance and was generally replaced by clade 3 at high relative abundance, whereas clade 1 proportions appeared more random but generally occur at low levels. This pattern intensified in the high A-lineage ancestry bin, although a few ‘pure’ clade 1 infections were seen at the lowest AmFV relative abundances.

**Figure 6:**
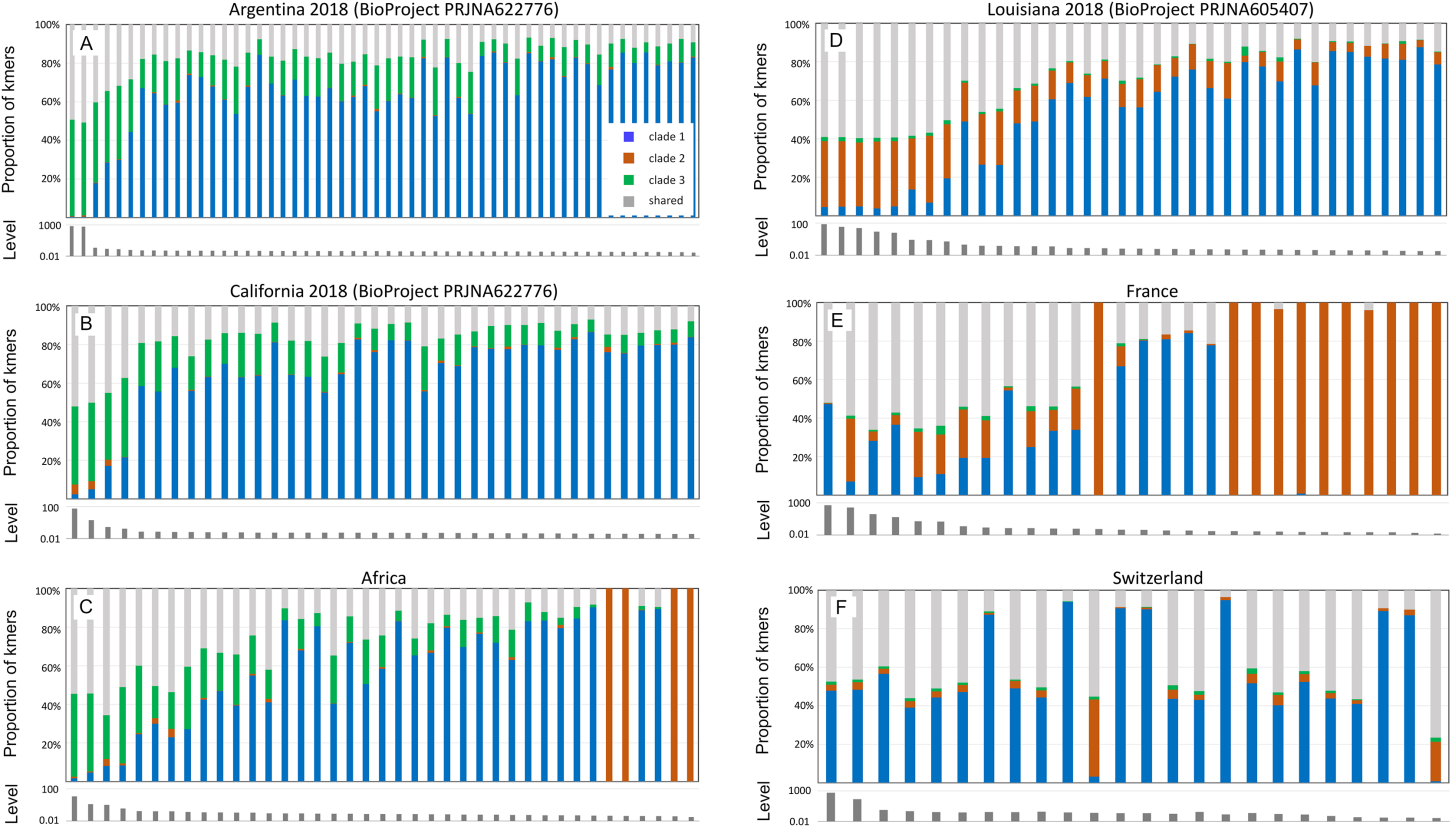
Apis mellifera filamentous virus (AmFV) clades show distinct regional distributions. Each panel contains a barplot of AmFV proportions in accessions and a barplot of the total abundance (level) of AmFV kmers relative to host kmers in those accessions. Accessions are from the same BioProject and year where noted, and are sorted by total AmFV abundance. Panels (A–C) are samples and geographic regions in which hosts have high A-lineage ancestry, whereas panels (D–F) are samples and geographic regions in which hosts have low or no A-lineage ancestry.

Figure 5 pools infection data without regard to spatial or temporal factors, yet A-lineage ancestry has a distinct and dynamic geographic distribution in the Americas (Calfee et al., 2020). Examining the proportions of AmFV clades in specific regions may therefore reveal additional details of host-pathogen genotypic variation (Fig. 6). For example, AmFV clade proportions are strikingly similar between samples from Africa and samples from feral honey bees collected by Calfee et al. (2020) along African-European honey bee hybrid zones in the Americas (BioProject PRJNA622776). In those data (Fig. 6), clade 3 AmFV replaces clade 1 AmFV as relative AmFV abundance increases in hosts. In contrast, a study of U.S. commercial bees (BioProject PRJNA605407) revealed a distribution of AmFV clades more similar to that found in western European countries such as France and Switzerland. In those samples, clade 3 is effectively absent whereas clade 1 and clade 2 are found in both mixed and pure infections. In the US commercial-bee samples, clade 2 replaces clade 1 as overall AmFV abundance increases, but this does not consistently occur in the European samples. Note that as the number of sampled kmers increases, the ratio of clade-specific to shared AmFV should approach 1:1 if a single clade is predominant. This generally holds true in Fig. 6, indicating that kmer sampling is approximately unbiased with respect to AmFV clade of origin despite the small number of genomes used to define them.

Although geolocated data from the eastern US and much of Europe are limited in the dataset (*i.e*., latitude and longitude were often not explicit in the BioSample metadata), the current results suggest that clade 3 arrived in the Americas with the well-known introduction of African honey bees to Brazil and subsequent expansion of A-lineage ancestry. This conclusion is further supported by a small number of positive samples from California collections spanning several decades (BioProject PRJNA385500; Fig. S8). In these wild-collected bees, AmFV clade proportions were similar to the European and US commercial bee data sets in Fig. 6 until approximately the late 1990s, at which point clade 3 AmFV is detected and AmFV clade mixtures become more variable. In fact, this BioProject is one of three in Table 1 that includes genetically admixed hosts and also exhibits a high rate of AmFV infection. While other factors may contribute to this pattern, such as vertical transmission or shared environment in the hybrid cross represented by BioProject PRJNA592197, the results are also consistent with the hypothesis that disrupted associations between co-evolved hosts and pathogens in hybrid zones promotes elevated infection rates.

### AmFV detection and relative abundance in Varroa DNA accessions

While AmFV has previously been detected in DNA extracted from *V. destructor*, such surveys do not discern whether AmFV replicates in *V. destructor* or is only passively acquired during feeding by *V. destructor* on honey bee hosts. Since both AmFV and *A. mellifera* kmers are detectable in *V. destructor* DNA accessions as a result of parasitic feeding (File S1), their relative abundance could potentially address this question. With the same filters applied (see Methods), detection rates in *V. destructor* (294 of 1,541 positive) and *A. mellifera* DNA accessions (396 of 2,222 positive) did not differ by contingency test overall (Fisher’s exact test, *P* = 0.346). However, high numbers of AmFV kmers were sometimes found in a *V. destructor* sample when *A. mellifera* kmers were rare or absent (Fig. S9A). For example, accession DRR292752, from a single female Varroa mite sample from Illinois, had 81,479 total AmFV kmer counts but only seven *A. mellifera* kmer counts, a strong outlier relative to other accessions (circled in Fig. S9A). Furthermore, relative abundances of AmFV to *A. mellifera* appeared higher in *V. destructor* accessions than in *A. mellifera* accessions on average when the threshold for inclusion (total kmer counts) was low (Fig. S9B). The latter relationship appears to be explainable by ascertainment bias, as AmFV is more likely to be detected at low search efforts when hosts have high infection levels. When minimum thresholds of *A. mellifera* kmer counts were imposed (*i.e*., when greater sampling effort was required), the apparently greater AmFV relative abundance in *V. destructor* disappeared (Fig. S9C). While AmFV infection levels in honey bee hosts several orders of magnitude above the median would seem to be required to explain the highest kmers counts observed in Varroa accessions, outliers of this scale are in fact observed in honey bee accessions (Figs. S9A and S9C). I conclude that AmFV kmer counts from *V. destructor* accessions appear to be consistent with passive acquisition from *A. mellifera* hosts, although more investigation is warranted.

### Long AmFV transcript contigs originate or terminate near tandem repeats

Transcriptome assembly was performed for 63 RNA accessions that were above absolute and relative thresholds of AmFV abundance (see Methods). The objective of this analysis was to identify whether long multicistronic transcripts are recovered (as reported by Moldován et al., 2018 for a baculovirus) and to characterize genomic features associated with transcript boundaries. For twelve accessions, the longest transcript contig was greater than 50 kb (File S8); nine of these had edges within or adjacent to tandem repeats of 8–13 bp (Fig. S10). Contig edges do not themselves demonstrate transcription start sites, as contigs may be incomplete and their orientation is not explicitly known (they encompass ORFs in both directions). Nonetheless, the frequency of the pattern and the recovery of transcripts that begin or end in the same location in multiple accessions (Fig. S10) suggest a role of such repeats in either transcript initiation or processing. Termination of long contigs at or near repeats is unlikely to be an artifact of assembly *per se* (*i.e*., an inability of the algorithm to traverse repetitive regions), as repeats of this length are generally short enough to be bridged by single reads and many low-complexity regions occur throughout the genome and are contained within transcripts of this length (see Fig. 3B). Similar tandem repeats occur in baculoviruses and nudiviruses and are believed to have roles in replication or transcription in those taxa (Wang et al., 2007; Williams, Bergoin & van Oers, 2017).

### Relative abundance of AmFV ORFs in RNA data varies by tissue

The relative abundance of reads mapped to annotated ORFs revealed different expression profiles among tissues (Fig. S11). A spearman correlation matrix of (log-ratio transformed) ORF relative abundance indicates that AmFV expression in brain or head tissue and certain abdominal organs (*e.g*., the sting gland and Nasonov’s gland) tend to be distinct from whole-body tissue profiles. Since biological and technical factors that could contribute to variation in expression typically vary less within BioProjects due to shared methodology, I selected SRA accessions from two individual BioProjects containing multiple positive samples in order to more directly contrast brain *vs* whole-body expression. For this extended analysis, I included additional full accessions from these BioProjects by suspending the AmFV abundance thresholds originally required for assembly. The two BioProjects compared were PRJNA706851, a metatranscriptome survey of whole-body tissue from China (Li et al., 2023), and PRJNA740269 (Tsvetkov & Zayed, 2021), an analysis of gene expression in honey bee brains.

**Figure 7:**
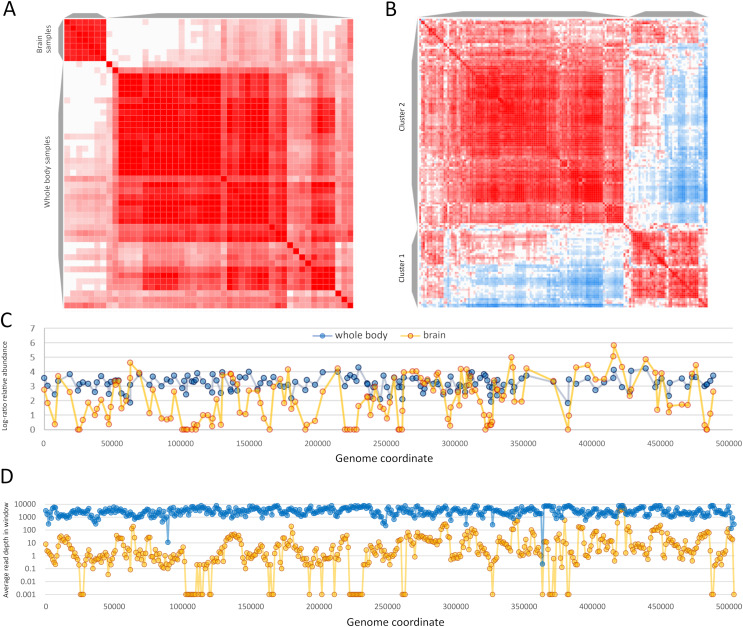
Apis mellifera filamentous virus (AmFV) exhibits contrasting expression patterns in whole body and brain tissue. (A) Sample-level similarity matrix based on open reading frame (ORF) relative abundance levels in RNA accessions. The strength of shading increases with increasing pairwise similarity. Two distinct clusters were computed corresponding to whole body and to brain accessions. (B) ORF-level similarity matrix across samples, with two distinct expression clusters recognized (see text for details). (C) Relative expression of ORFs in whole body and brain accessions, using log-ratio transformed values (see text for details). Points indicate calculated values and lines indicate genomic trends. (D) Read coverage in 1-kb genomic windows in the same RNA accessions, averaged across all accessions in each BioProject. Points indicate calculated values and lines indicate genomic trends.

Sample-level clustering from these two BioProjects differentiated brain from whole-body samples more strongly than the set of 63 samples above (Figs. 7A and 7B). ORF-level clustering identified a set of ORFs that were effectively unexpressed in brain, *i.e*., no or minimal expression was detected (Fig. 7C). Coverage in consecutive genomic windows of 1 kb closely parallels that of the ORF-specific analysis (Fig. 7D), and several additional genomic windows lacking RNA coverage in the brain data set do not contain annotated ORFs (*e.g*., near genomic positions 200 and 365 kb). ORFs that were undetected in brain occur in multiple genomic regions but several distinct clusters are evident and include ORFs in opposite orientations (File S7). RNA coverage appears more variable overall in the brain accessions compared to the whole-body accessions, with more frequent shifts in transcript abundance across genomic windows (Fig. 7D). ORFs in the ‘brain-off’ expression cluster (labeled “Cluster 1” in Fig. 7A**)** do not obviously differ in ω or ENC’ compared to other ORFs (see File S7). Peak expression in these brain accessions was observed for the ORF pair gp210 and gp211 (based on their annotated coordinates as well as the encompassing 1 kb genomic windows); these ORFS have no recognized domain and include repetitive tracts. Genomic coverage and ORF-level relative abundance used in these analyses are available in File S9.

In contrast to DNA accessions, in which individual BioProjects often represented large surveys of natural genetic variation, RNA BioProjects contained smaller numbers of accessions typical of RNA-Seq experiments (17.7 on average, range 1–171). Manual review of BioProject summaries enabled more complete metadata classifications of positive accessions so that an explicit comparison of AmFV relative abundance in head tissue *versus* abdomen tissue could be made in female workers (File S10), with whole body accessions excluded. I downsampled one large BioProject (PRJNA521949, a study of exposure to the pesticide imidacloprid (Tsvetkov & Zayed, 2021)), to only include untreated control samples. The final data set contained 78 accessions from 28 BioProjects. The proportion of total kmers that were classified as AmFV in these accessions was then modeled as a binomial regression with tissue and BioProject as predictors and combined AmFV and *A. mellifera* kmer counts as weights. While BioProject was (not unexpectedly) a much stronger predictor, tissue type was also significant (*P* = 3.26E−5), indicating lower levels of AmFV RNA in head tissue than abdomen relative to host (File S10).

## Conclusion

AmFV is commonly detected in molecular surveys of honey bee diseases and more than one in five DNA accessions were AmFV positive in this analysis using what are likely conservative criteria relative to sensitive PCR assays (cf. Hartmann et al., 2015). Despite its prevalence, AmFV has remained enigmatic with respect to ecology, genetics, and economic impact. Not least among the uncertainties is the actual complement of ORFs that AmFV expresses, particularly given that criteria for annotating hypothetical ORFs in single genome sequences are somewhat arbitrary, such as minimum ORF length and the extent to which repetitive or compositionally biased regions should be masked. Single reference genomes may also have errors that obscure valid ORFs. The availability of new reference genomes (Yang et al., 2022) and the extraction of consensus genomes from public data (this study) allow annotations to be buttressed by principles of molecular evolution. Here I identified evolutionary support for a subset of previously annotated ORFs based on conservation of coding frame, low evolutionary rates, and biased codon usage consistent with a functional gene. Furthermore, the 47 ORFs Yang et al. (2022) validated by proteomics provides an independent means of determining the ranges of ω and ENC’ to be expected for AmFV structural proteins, and these were indeed found to be evolutionarily conserved (Fig. 4). Most of the remaining 108 conserved ORFs analyzed had ENC’ and ω values overlapping those validated by proteomics. However, there remain numerous ORFs for which data are insufficient to evaluate evolutionary conservation at present, such that the total number of validated ORFs is likely to grow with additional study. It also remains possible that ORFs rejected here are functional within certain clades and have become pseudogenes in other clades. A few ORFs had high ω indicative of neutral evolution, but the functional implication of these values should be interpreted cautiously. The total number of substitutions in the six-genome alignment was often small, making the relative rate of synonymous and nonsynonymous substitutions challenging to accurately estimate.

Note that while Yang et al. (2022) describe some ORFs annotated by Gauthier et al. (2015) as “absent” from accession OK392616.1, these differences are only at the level of feature annotation (primarily based on different length or homology criteria) rather than actual loss of orthologous sequence (as evidenced by reciprocal BLASTN, for example—see File S11). Additionally, while the AmFV genome is represented linearly here for simplicity, it remains unclear to what extent linear *vs* circular forms occur. Gauthier et al. (2015) provided PCR evidence of genome circularization, and viral taxa for which AmFV shows phylogenetic affinity, such as Nudiviridae, Baculoviridae, and Hytrosaviridae (Gauthier et al., 2015, Yang et al., 2022), are also characterized by circular genomes (Hulo et al., 2011). In contrast, Yang et al. (2022) proposed a linear genome based on a lack of sequence data bridging the termini of their assembly. However, that argument presumes an absence of sequence-capture biases (such as extreme nucleotide composition or secondary structure) despite a reasonable expectation that such could occur in AmFV. It should also be noted that the genome assembly recovered by those authors is truncated at the 3′ end by approximately 3 kb in the linear alignment compared to the five other sequences used in this study (File S3), which could explain why no reads bridged the assembly termini.

Assembled transcripts and co-expression patterns are consistent with long, multicistronic transcripts containing ORFs in opposing directions, similar to the results of Moldován et al. (2018). While it is possible that overlapping but distinct transcripts could be erroneously assembled into a larger, artifactual contig, this would require long untranslated regions as the gap between annotated ORFs averages more than a kilobase and is often substantially greater. Thus, further analysis of AmFV transcripts with long-read or strand-specific sequencing would be desirable to further confirm these results.

A distinct expression pattern in the brain was revealed by a general comparison of all RNA accessions above a threshold level of AmFV abundance as well as a more targeted comparison contrasting two large BioProjects with numerous positive samples. The set of AmFV ORFs undetected in BioProject PRJNA740269 includes gp005, which has a metalloprotease domain suggesting a role in cleaving polypeptides, the RecB homolog (gp122) discussed above, and all ORFs encoding subunits of ribonucleotide reductase (gp114 and gp216/gp217). Note that gp216 and gp217 are likely a single ORF that has been split around a frameshift in reference genome NC_027925.1, as the protein domain is contiguous across the two ORF annotations, which themselves overlap by 17 bases (see https://www.ncbi.nlm.nih.gov/gene/?term=APL35_gp216). Ribonucleotide reductase is the rate-limiting step in the conversion of RNA to DNA and many DNA viruses induce either host- or viral-encoded ribonucleotide-reductase activity that is required for replication (*e.g*., Cameron et al., 1988). If this holds for AmFV as well, this expression pattern suggests that AmFV may not fully complete its lifecycle in brain tissue but nonetheless accumulates at detectable levels and expresses the majority of its genome. However, it bears noting that several examples are known of viral-encoded ribonucleotide reductase subunits with novel and non-essential functions (*e.g*., Lembo & Brune, 2009). An important follow-up to these results would be to evaluate differential expression of the host transcriptome in infected brain tissue, as it is possible AmFV manipulates the host physiologically or behaviorally to benefit AmFV transmission overall, as has been documented in other insect-virus interactions (*e.g*., Ikeda, Hamajima & Kobayashi, 2015; Blanc & Michalakis, 2016).

Coevolution between AmFV and *A. mellifera* is suggested by the distinct geographic distributions of AmFV clades and the dependence of their relative abundance on host ancestry (Figs. 5–6). Interestingly, all clades were found to occur at high relative abundances in hosts lacking A-lineage ancestry, which presumably represents mostly managed bees with predominantly European-domesticate ancestry (see Momeni et al., 2021). In contrast, only clade 3 was found to achieve high abundances in hosts with high A-lineage ancestry. It is possible that this asymmetry reflects demographic disequilibrium between host and pathogen lineages brought about by global trade in honey bees and honey bee products rather than deterministic differences in virulence. Experimental tests of host-lineage interactions are needed to assess whether host-virus associations are deterministic or have environmental covariates, and how such interactions are mediated. Fine-scale analysis of natural hybrid zones between A-lineage and non-A lineage honey bees in the Americas (*e.g*., Calfee et al., 2020) could provide an additional means of identifying genetic loci that influence host-pathogen interactions. Furthermore, several other distinct lineages of honey bees have been documented (Wallberg et al., 2014; Momeni et al., 2021) that cannot be distinguished by the methods used here, but which may also exhibit lineage-specific dynamics.

While data submitters should be commended for providing valuable metadata for their sampling and sequencing methodology, including many voluntary fields specific to their studies, it remains challenging to aggregate those metadata across different BioProjects due to a lack of consistent vocabulary. In this study, I defined three metadata categories that could be frequently inferred from submitted field names and contents (“inferred sex”, “inferred developmental stage”, and “inferred tissue”; File S1), yet the proportion of accessions that were undetermined for these bins in DNA accessions was 1.5%, 51.3%, and 22.2% respectively. An alternative field commonly used to place metadata of this nature is the “library title” field within the SRA metadata rather than in the BioSample, but that free-text field is not easily parsed. Surprisingly few BioSample accessions specified latitude and longitude (13.8%), which may reflect different privacy policies or approaches to sharing geolocation data among investigators and data contributors. I believe it would enhance the power of data sharing to address novel questions in honey bee biology if a more controlled vocabulary for metadata fields specific to this species was widely adopted.

## Supplemental Information

10.7717/peerj.16455/supp-1 Supplemental Information 1 A single pair of alternative kmers is a useful proxy of A-lineage ancestry that recapitulates the known distribution in honey bees. A. Aligned reference accessions of 18S ribosomal rRNA sequence of honey bee subspecies, in which*Apis mellifera capensis*and*A. mellifera scutellata*are African subspecies and*A. mellifera mellifera*and*A. mellifera ligustica*are European subspecies. The box outlined in yellow indicates the kmers selected to distinguish A-lineage hosts from non-A-lineage hosts. B. Relative abundance of alternative 18S kmers correlates with kmers derived from nuclear SNPs that discriminated A-lineage hosts in a previous study (see text for details). C. Proportion of 18S kmers that are A-lineage derived in accessions matches expected dominance in Africa, expected absence in Europe, and known clines of A-lineage ancestry in South America and North America.

10.7717/peerj.16455/supp-2 Supplemental Information 2 Abundances of randomly selected kmers are broadly representative of the Apis mellifera filamentous virus (AmFV) genome. A. For individual DNA accessions with at least 10,000 counts of AmFV kmers, the relative abundances of kmers in 10-kb genomic windows are plotted as points with lines added to illustrate trends. B. Points represent the coefficient of variance (CV) across all accessions in panel A in each window, with lines added to illustrate trends.

10.7717/peerj.16455/supp-3 Supplemental Information 3 Different representations of Apis mellifera filamentous virus (AmFV) genomic diversity are all consistent with three major phylogenetic clades. Colored symbols represent the original reference genome and two additional genome sequences chosen to represent the novel clades of AmFV identified in this study. A. Phylogeny of consensus genome sequences generated by mapping DNA reads to a single reference genome. B. Phylogeny of six approximately full-length genome sequences, two from each of the three major clades in panel A. C. Phylogeny of consensus genome sequences generated by mapping DNA reads to the three marked genome sequences generated by *de novo*assembly in this study or previously. D. Phylogeny of accessions based on kmer distance only. For short-read accessions, kmers were counted in the original subsample downloaded for each, whereas for NC_027925.1 they were counted from the reference sequence itself.

10.7717/peerj.16455/supp-4 Supplemental Information 4 The number of homopolymers of each nucleotide varies across the Apis mellifera filamentous virus genome in a pattern closely matching the overall nucleotide composition bias. The horizontal axis represents 500-bp windows along the genome reference, and the vertical axis represents the number of homopolymers of length five or more in these windows.

10.7717/peerj.16455/supp-5 Supplemental Information 5 Apis mellifera filamentous virus (AmFV) open reading frames (ORFs) have stronger codon bias than host coding sequence on average. In both panels, the horizontal axis represents the codon bias score ENC’, which has a theoretical maximum of 61 (the number of codons specifying amino acids in the standard genetic code), with lower values representing increasingly biased use of codons. ENC’ is measured relative to a reference set of coding sequences (see text for details). A. Histogram of codon bias scores (horizontal axis) for AmFV ORFs relative to a set of bee ribosomal protein genes, which are presumed to be highly expressed based on their functional role. B. Histogram of codon bias scores (horizontal axis) for Apis mellifera coding sequences relative to the same reference set of ribosomal protein genes.

10.7717/peerj.16455/supp-6 Supplemental Information 6 Population genomic statistics calculated in sliding windows for each Apis mellifera filamentous virus (AmFV) clade. Points represent calculated values for each window, whereas lines are included to represent trends only. Each line is a different length because the reference genomes vary in length. A. Nucleotide diversity calculated in consecutive 10-kb genomic windows. B. Tajima’s D calculated in consecutive 10-kb genomic windows.

10.7717/peerj.16455/supp-7 Supplemental Information 7 Localized genomic variation in the *de novo* assembly of accession SRR5580859 is consistent with recombination with an unknown, highly divergent Apis mellifera filamentous virus (AmFV) lineage. A. Extensive synonymous and nonsynonymous variation within open reading frame (ORF) gp236, including indels but with no disruption of coding frame, in an otherwise almost invariant alignment. B. Alignment of genomic sequence showing SRR5580859 to have many unique polymorphisms in the initial part of the alignment but sharing most polymorphisms with the other clade 2 reference sequence (SRR15173571) in the latter part of the alignment. The inferred breakpoint consistent with recombination is inferred to be within the boxed region. C. Neighbor-joining gene trees for the six terminal ORFs of the AmFV genome, extracted from six assembled and aligned reference genomes. Sequences from the same clade are marked accordingly. The magnitude of divergence for accession SRR5580859 is reflected in the branch lengths (note differences in scale bars among gene trees).

10.7717/peerj.16455/supp-8 Supplemental Information 8 Apis mellifera filamentous virus (AmFV) clade proportions in positive honey bee accessions collected before and after the introduction of A-lineage ancestry to that state. The year of original collection of the sequenced sample is noted on the horizontal axis, and accessions are sorted temporally. Asterisks indicate that A-lineage kmers were detected in the accession. See text for details.

10.7717/peerj.16455/supp-9 Supplemental Information 9 Apis mellifera filamentous virus (AmFV) kmer abundances in*Varroa destructor*DNA accessions appear consistent with a lack of replication in that species. A. Points represent total AmFV kmer counts for AmFV-positive accessions in*V. destructor*and A. mellifera, as a function of totalApis melliferakmer counts in those accessions. Some Varroa accessions have high AmFV kmer counts in the absence of any detected A. mellifera kmers. The circled point is a strong outlier of AmFV kmer counts discussed in the text. B. High ratios of AmFV kmers to A. melliferakmers were found for some*V. destructor*accessions, but only when the total A. mellifera counts were low. C. Imposing minimum thresholds on the number of A. mellifera kmer counts in each*V. destructor*accession indicates that with adequate sampling effort, no evidence is found of*V. destructor*accessions with higher AmFV relative abundances than the hosts that they parasitize.

10.7717/peerj.16455/supp-10 Supplemental Information 10 The boundaries of long transcript contigs are strongly associated with tandem repeats in the Apis mellifera filamentous virus (AmFV) genome. Four examples of genomic locations in which contigs greater than 50 kb begin or end near a tandem repeat. In each case, one or more contigs are shown aligned to reference genome NC_027925.1. The contig name is that generated by the assembly program, preceded by the accession identifier.

10.7717/peerj.16455/supp-11 Supplemental Information 11 Apis mellifera filamentous virus (AmFV) expression profiles tend to cluster by tissue type. Each cell of the matrix represents the pairwise Spearman correlation coefficient between 63 RNA accessions with AmFV abundances above minimum thresholds. Accession IDs are listed along with the tissue analyzed, as specified in the associated metadata, not as binned in this study (see text for details). Positive correlations are shaded blue and negative correlations are shaded red, as indicated by the legend. Samples are sorted by hierarchical clustering using Ward’s method. The underlying expression values for ORFs in samples were transformed using a log-ratio approach (see text for details).

10.7717/peerj.16455/supp-12 Supplemental Information 12 Metadata and count data for scanned Sequence Read Archive (SRA) run accessions. Runs are discrete sequencing sets produced by a sequencing platform and are grouped with related runs under a BioProject. BioSamples are accessions linking metadata for a biological sample that was sequenced; only a subset of user-provided metadata are shown that are relevant to the analyses performed in this study. Run and sample metadata are as provided by the National Center for Biotechnology Information (NCBI), see www.ncbi.nih.nlm.gov for more information. Derived metadata are sample metadata reassigned to simplified categories for this study, and are therefore subject to error or alternative interpretation. Count data are raw counts of kmer abundances in a subsample of reads (see text for details). Note that the column headers are not the same for each page.

10.7717/peerj.16455/supp-13 Supplemental Information 13 Kmers of length 31 that were extracted from reference sequences as described in the text that were used to identify exact matches in Sequence Read Archive run accessions. Each page lists a different set of kmers that were used to quantify different classess of sequence.

10.7717/peerj.16455/supp-14 Supplemental Information 14 Multiple sequence alignment in FASTA format of two reference AmFV accessions and four AmFV genome assemblies generated by this study.

10.7717/peerj.16455/supp-15 Supplemental Information 15 Multiple sequence alignments in FASTA format of ORFs extracted from the six-genome alignment based on annotated coordinates for NC_027925.1. 241 concatenated alignments, grouped by ORF name and secondarily by a label indicating whether the ORF appears to be evolutionarily conserved in the six genomes, *i.e*., “passed”, “failed”, or “indeterminate”.

10.7717/peerj.16455/supp-16 Supplemental Information 16 Alignment of 38 AmFV consensus genomes with reference accession NC_027925.1 used for phylogenetic analysis.

10.7717/peerj.16455/supp-17 Supplemental Information 17 Tandem repeats identified in AmFV genome reference NC_027925.1.

10.7717/peerj.16455/supp-18 Supplemental Information 18 Summary of ORF conservation and characteristics. See text for explanation and methodology. Any use of trade, firm, or product names is for descriptive purposes only and does not imply endorsement by the U.S. Government.

10.7717/peerj.16455/supp-19 Supplemental Information 19 Longest contig in each assembled RNA accession that exceeded 50 kb in length, in FASTA format.

10.7717/peerj.16455/supp-20 Supplemental Information 20 Log-ratio transformed relative abundance of each open reading frame (ORF) and in 1-kb genomic windows in 42 sequence data accessions positive for Apis mellifera filamentous virus (AmFV). Values are derived from mapping reads to reference accession NC_027925.1. Columns colored yellow are whole-body tissue samples from BioProject PRJNA706851 and columns colored green are brain tissue samples from BioProject PRJNA740269. Note that six accessions from these BioProjects that were AmFV positive were excluded here due to low total counts, rendering them not useful for relative gene expression analysis.

10.7717/peerj.16455/supp-21 Supplemental Information 21 The ratio of kmers assigned to Apis mellifera filamentous virus (AmFV) and host in 78 RNA sequence read archive (SRA) accessions for which tissue category could be inferred from BioSample metadata or BioProject summaries. All samples are inferred from metadata to be female adult workers, although the number of individuals represented in each BioSample is not explicitly known.

10.7717/peerj.16455/supp-22 Supplemental Information 22 Reciprocal alignment scores between open reading frames (ORFs) annotated in NCBI accessions OK392616.1 and NC_027925.1. Alignment metrics are standard output of BLASTN tabular format. Any use of trade, firm, or product names is for descriptive purposes only and does not imply endorsement by the U.S. Government.

## Additional Information and Declarations

### DNA Deposition

The following information was supplied regarding the deposition of DNA sequences: The raw data for this study consisted of 5,585 existing public accessions of the Sequence Read Archive (SRA) that were downloaded in whole or part for this study, and are listed in the Supplemental Files. All derived sequence data produced by this analysis are included in the Supplemental Files because metagenomic assemblies of heterogenous samples are not suitable for submission to GenBank.

### Data Availability

The following information was supplied regarding data availability: All SRA accessions and their associated metadata, sequence alignments used in analyses, and raw kmer counts are available in the Supplemental Files.
