# Supplementary figures and images for: Data mining reveals tissue-specific expression and host lineage-associated forms of Apis mellifera filamentous virus
Source: PeerJ. 2023 Nov 14;11:e16455. doi: 10.7717/peerj.16455 (PMC10655722; doi:10.7717/peerj.16455)

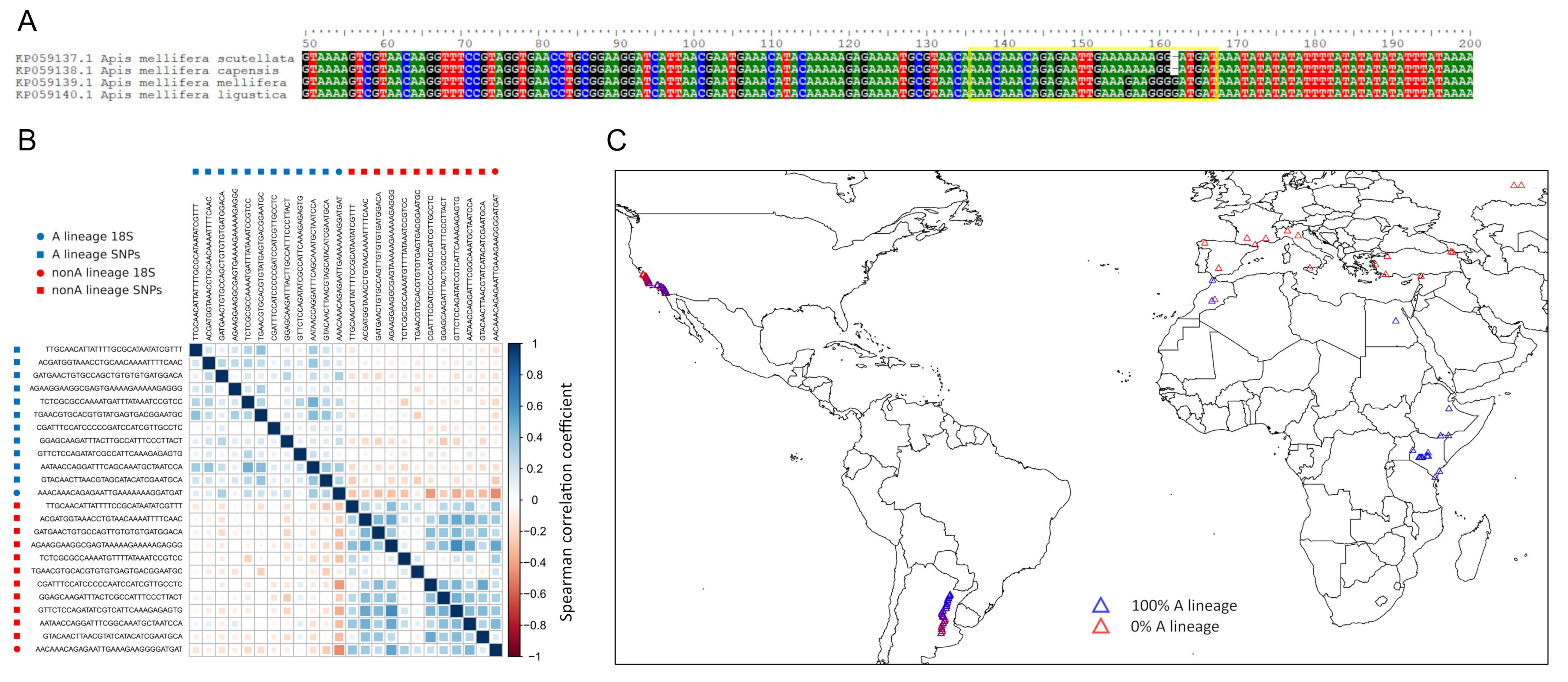

Supplement: Supplemental Information 1 — A. Aligned reference accessions of 18S ribosomal rRNA sequence of honey bee subspecies, in whichApis mellifera capensisandA. mellifera scutellataare African subspecies andA. mellifera melliferaandA. mellifera ligusticaare European subspecies. The box outlined in yellow indicates the kmers selected to distinguish A-lineage hosts from non-A-lineage hosts. B. Relative abundance of alternative 18S kmers correlates with kmers derived from nuclear SNPs that discriminated A-lineage hosts in a previous study (see text for details). C. Proportion of 18S kmers that are A-lineage derived in accessions matches expected dominance in Africa, expected absence in Europe, and known clines of A-lineage ancestry in South America and North America. [file peerj-11-16455-s001.png]

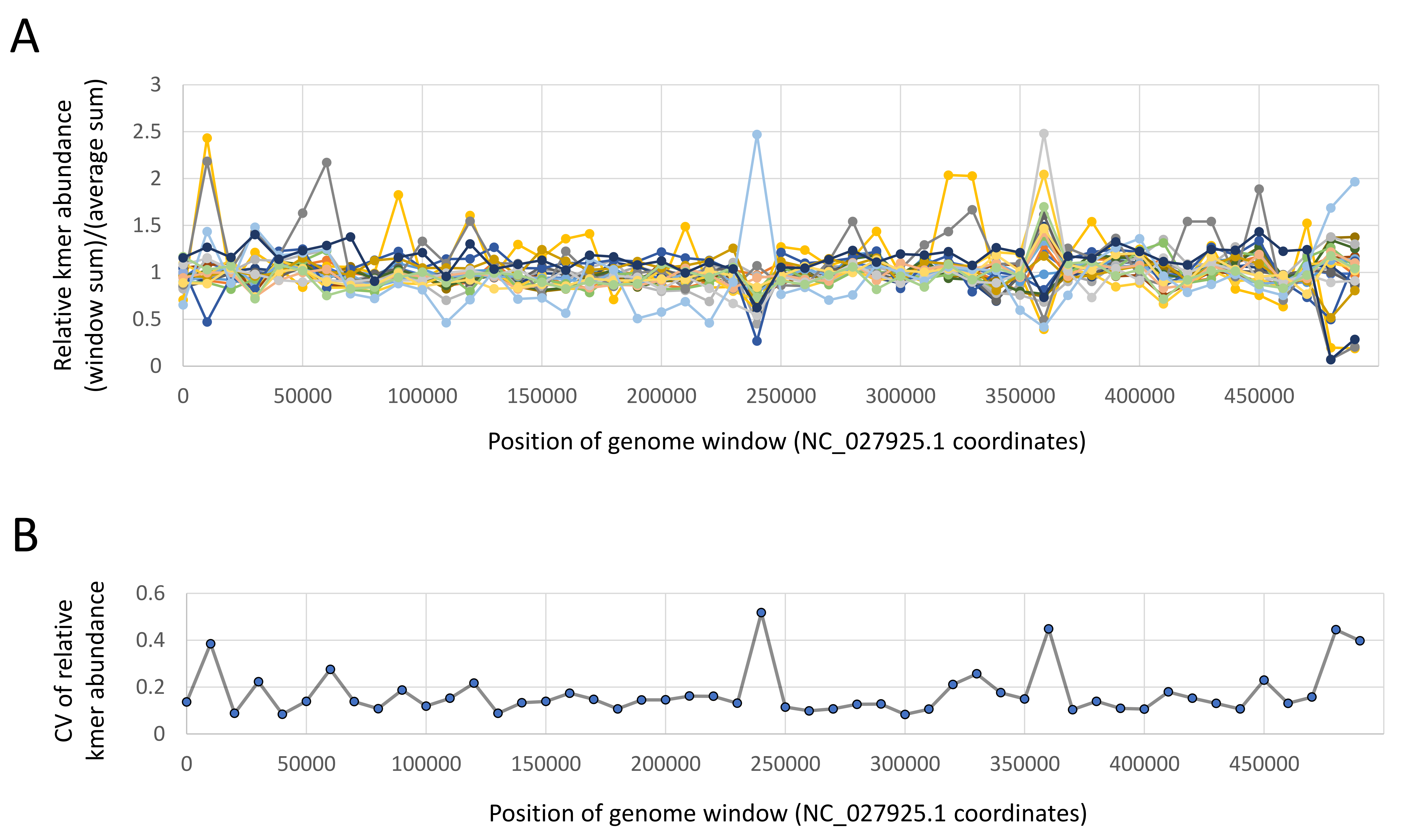

Supplement: Supplemental Information 2 — A. For individual DNA accessions with at least 10,000 counts of AmFV kmers, the relative abundances of kmers in 10-kb genomic windows are plotted as points with lines added to illustrate trends. B. Points represent the coefficient of variance (CV) across all accessions in panel A in each window, with lines added to illustrate trends. [file peerj-11-16455-s002.png]

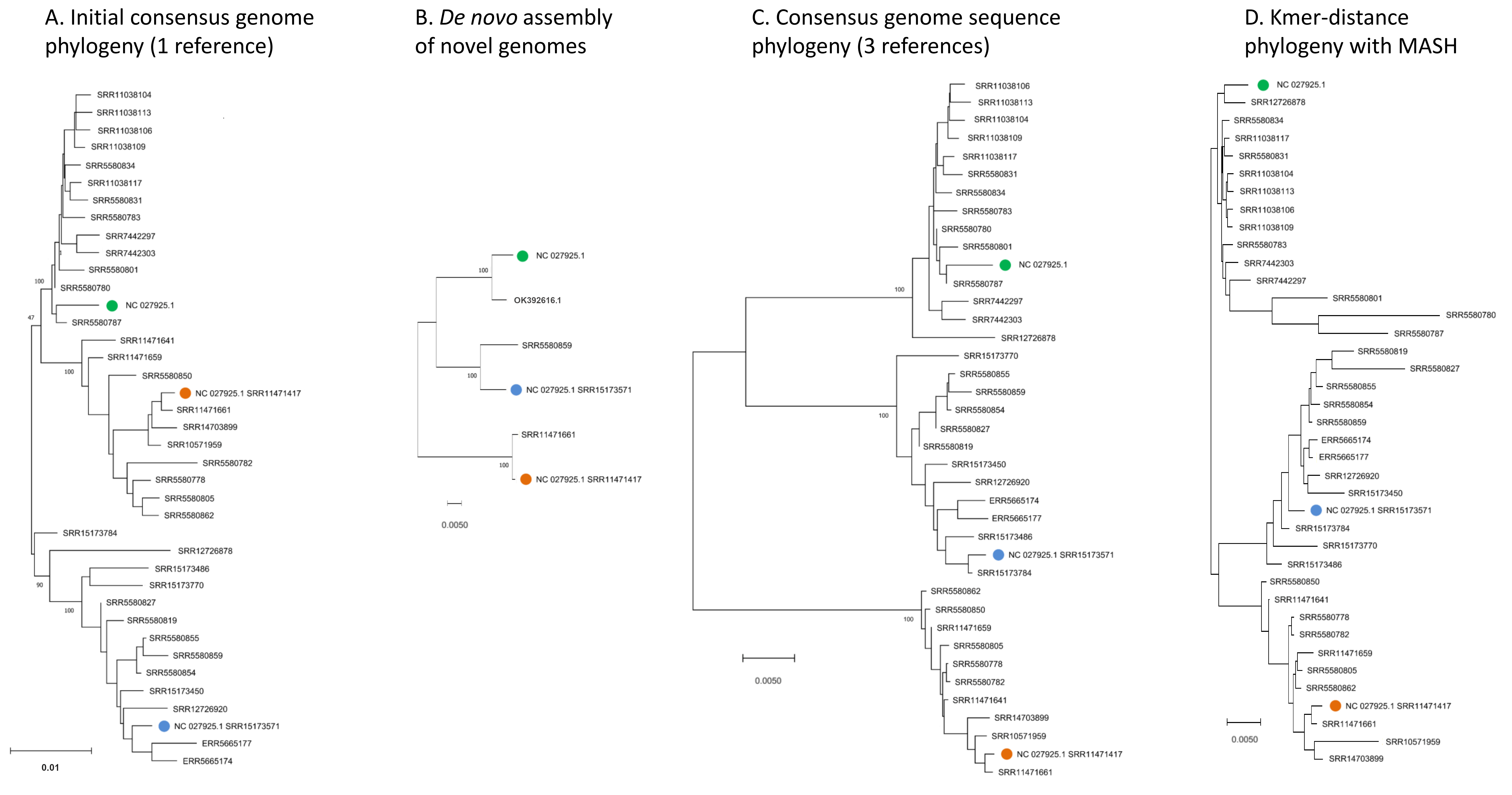

Supplement: Supplemental Information 3 — Colored symbols represent the original reference genome and two additional genome sequences chosen to represent the novel clades of AmFV identified in this study. A. Phylogeny of consensus genome sequences generated by mapping DNA reads to a single reference genome. B. Phylogeny of six approximately full-length genome sequences, two from each of the three major clades in panel A. C. Phylogeny of consensus genome sequences generated by mapping DNA reads to the three marked genome sequences generated by de novoassembly in this study or previously. D. Phylogeny of accessions based on kmer distance only. For short-read accessions, kmers were counted in the original subsample downloaded for each, whereas for NC_027925.1 they were counted from the reference sequence itself. [file peerj-11-16455-s003.png]

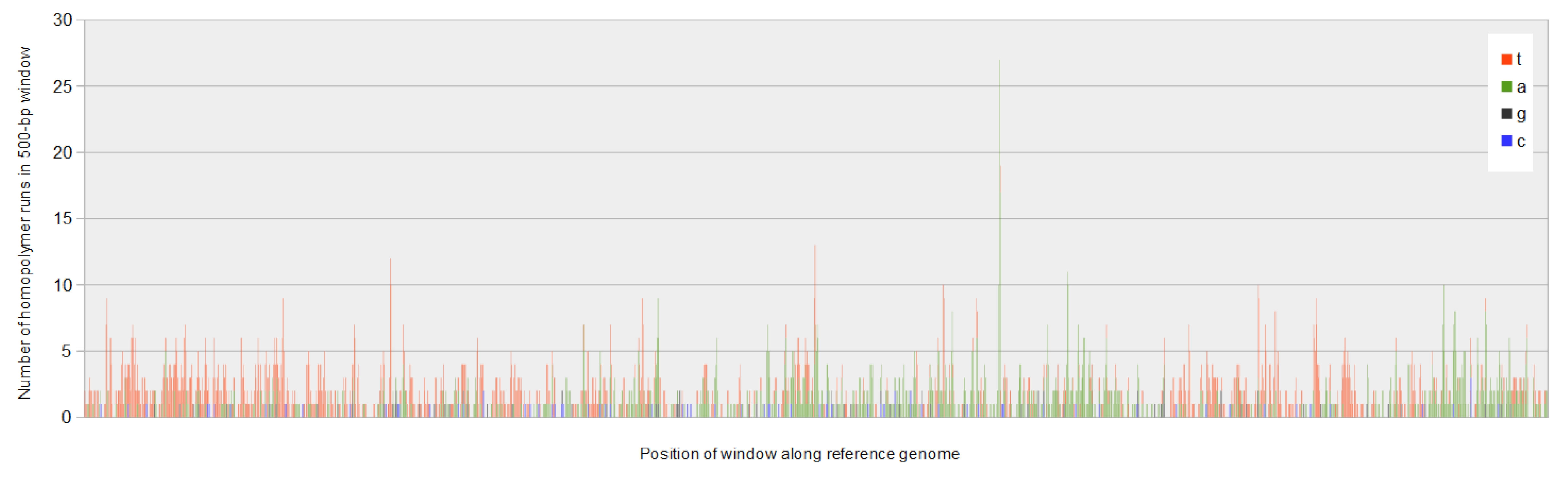

Supplement: Supplemental Information 4 — The horizontal axis represents 500-bp windows along the genome reference, and the vertical axis represents the number of homopolymers of length five or more in these windows. [file peerj-11-16455-s004.png]

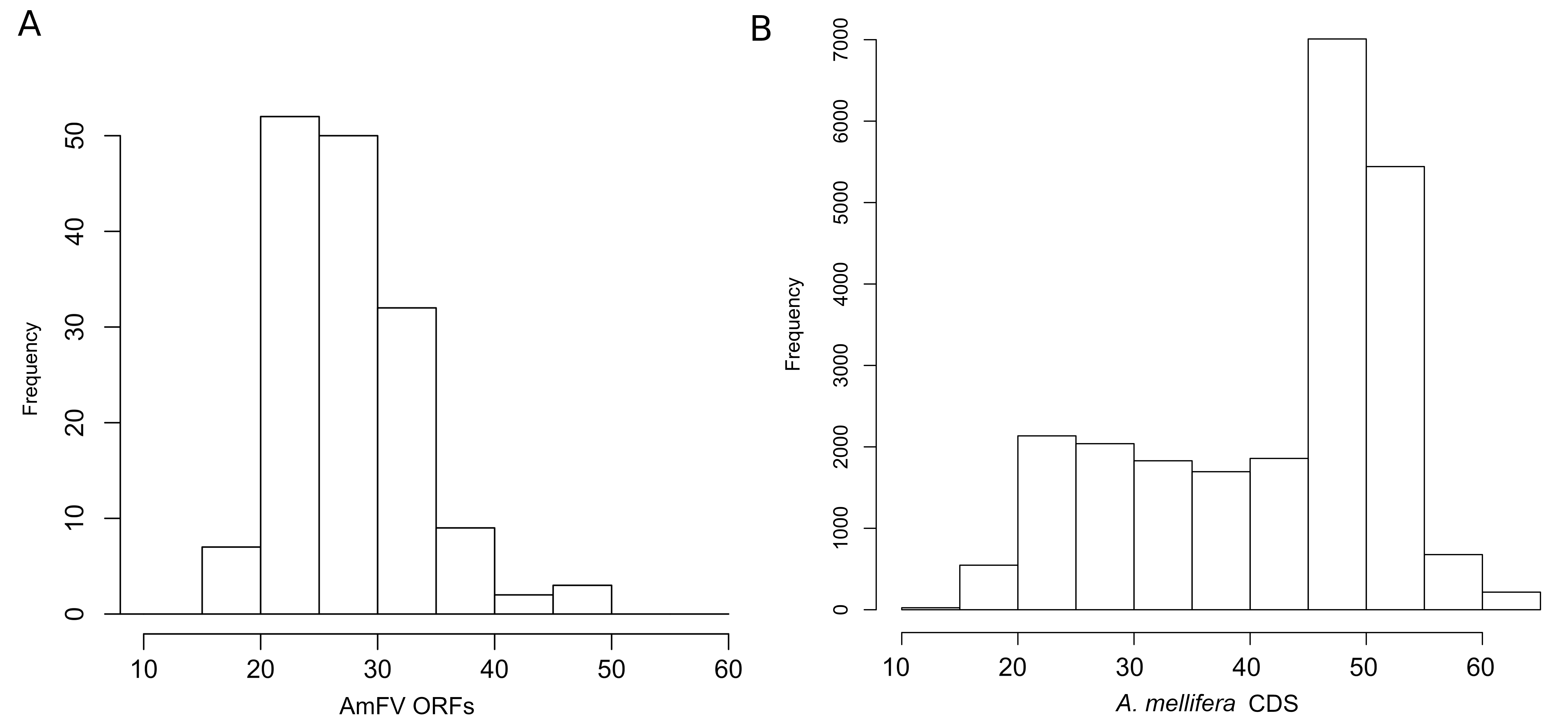

Supplement: Supplemental Information 5 — In both panels, the horizontal axis represents the codon bias score ENC’, which has a theoretical maximum of 61 (the number of codons specifying amino acids in the standard genetic code), with lower values representing increasingly biased use of codons. ENC’ is measured relative to a reference set of coding sequences (see text for details). A. Histogram of codon bias scores (horizontal axis) for AmFV ORFs relative to a set of bee ribosomal protein genes, which are presumed to be highly expressed based on their functional role. B. Histogram of codon bias scores (horizontal axis) for Apis mellifera coding sequences relative to the same reference set of ribosomal protein genes. [file peerj-11-16455-s005.png]

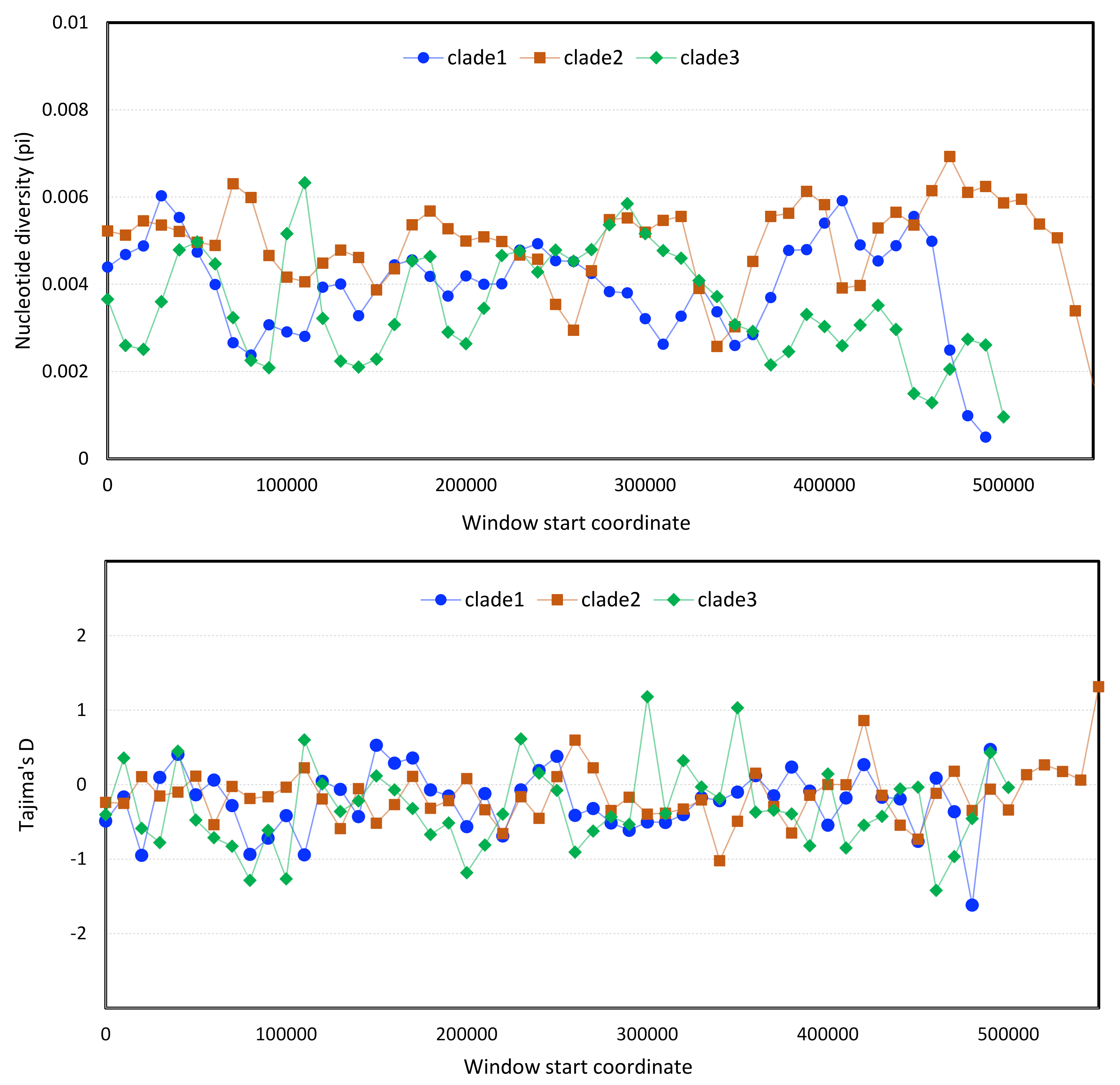

Supplement: Supplemental Information 6 — Points represent calculated values for each window, whereas lines are included to represent trends only. Each line is a different length because the reference genomes vary in length. A. Nucleotide diversity calculated in consecutive 10-kb genomic windows. B. Tajima’s D calculated in consecutive 10-kb genomic windows. [file peerj-11-16455-s006.png]

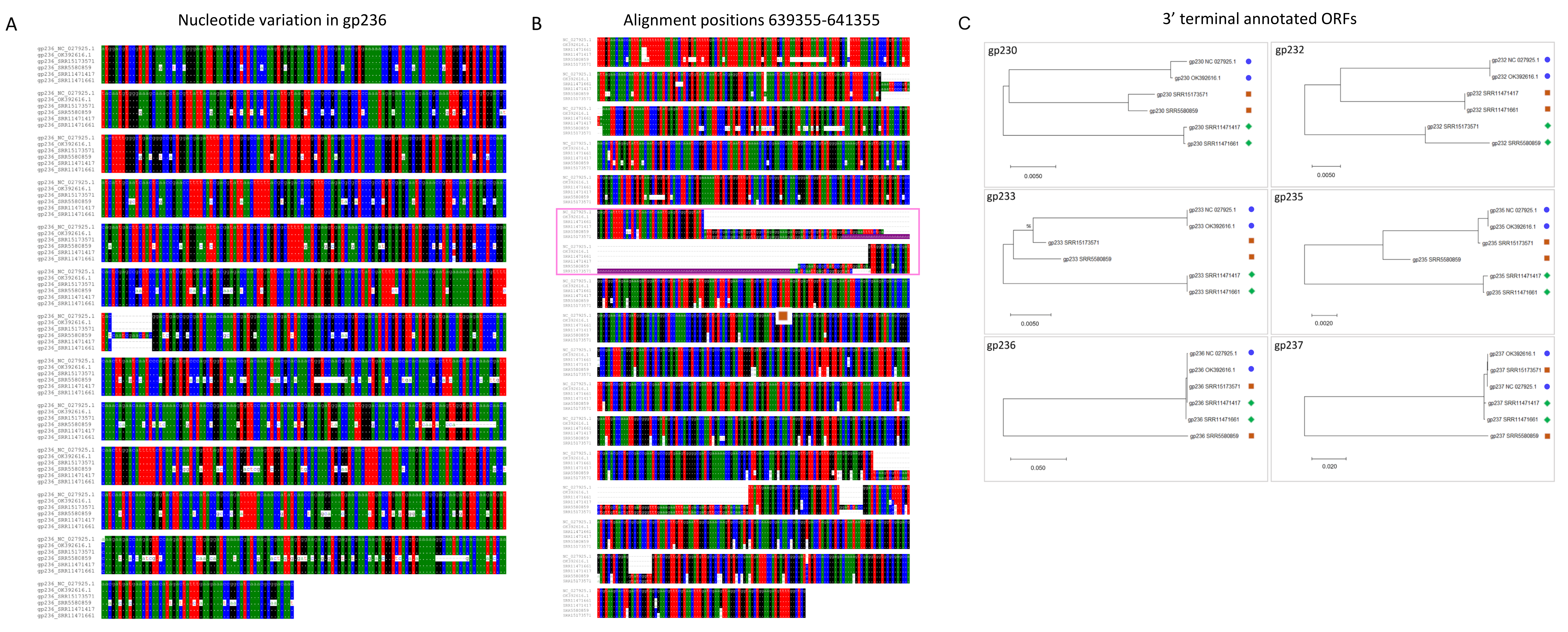

Supplement: Supplemental Information 7 — A. Extensive synonymous and nonsynonymous variation within open reading frame (ORF) gp236, including indels but with no disruption of coding frame, in an otherwise almost invariant alignment. B. Alignment of genomic sequence showing SRR5580859 to have many unique polymorphisms in the initial part of the alignment but sharing most polymorphisms with the other clade 2 reference sequence (SRR15173571) in the latter part of the alignment. The inferred breakpoint consistent with recombination is inferred to be within the boxed region. C. Neighbor-joining gene trees for the six terminal ORFs of the AmFV genome, extracted from six assembled and aligned reference genomes. Sequences from the same clade are marked accordingly. The magnitude of divergence for accession SRR5580859 is reflected in the branch lengths (note differences in scale bars among gene trees). [file peerj-11-16455-s007.png]

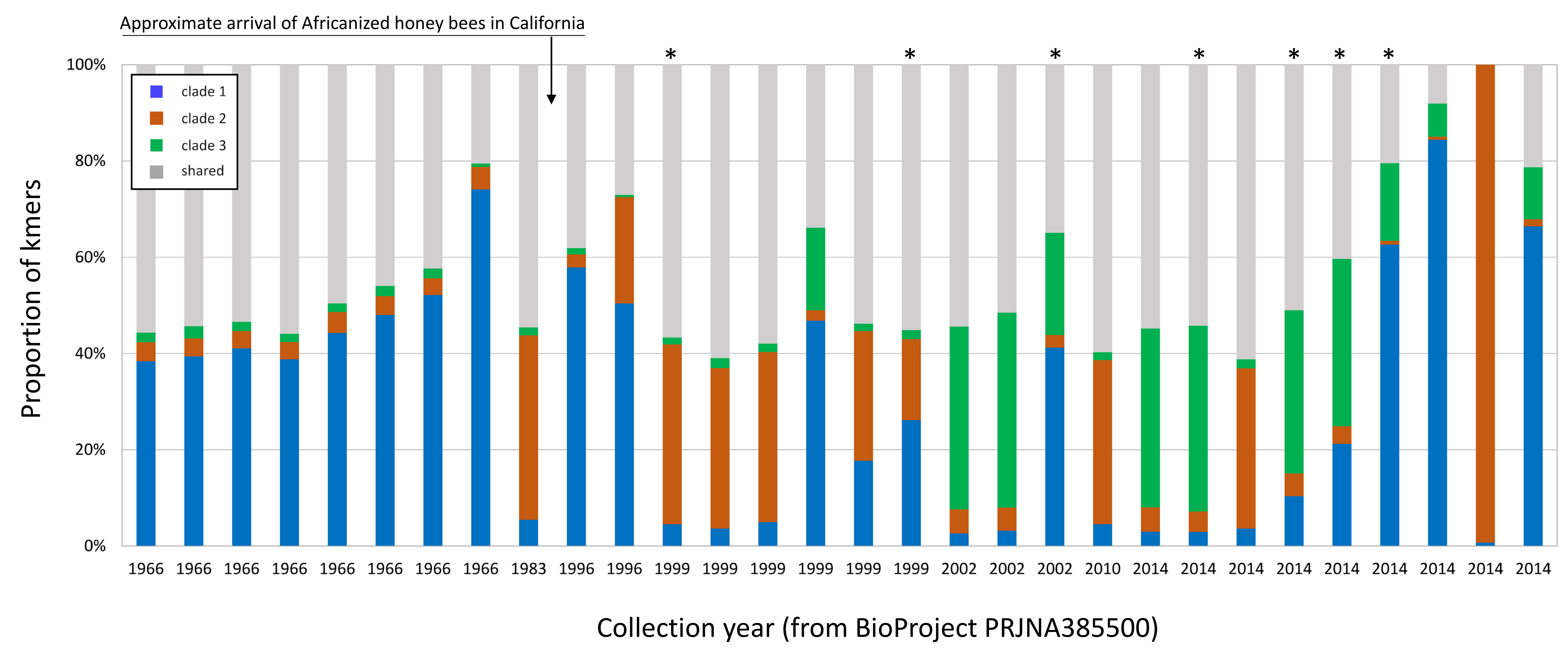

Supplement: Supplemental Information 8 — The year of original collection of the sequenced sample is noted on the horizontal axis, and accessions are sorted temporally. Asterisks indicate that A-lineage kmers were detected in the accession. See text for details. [file peerj-11-16455-s008.png]

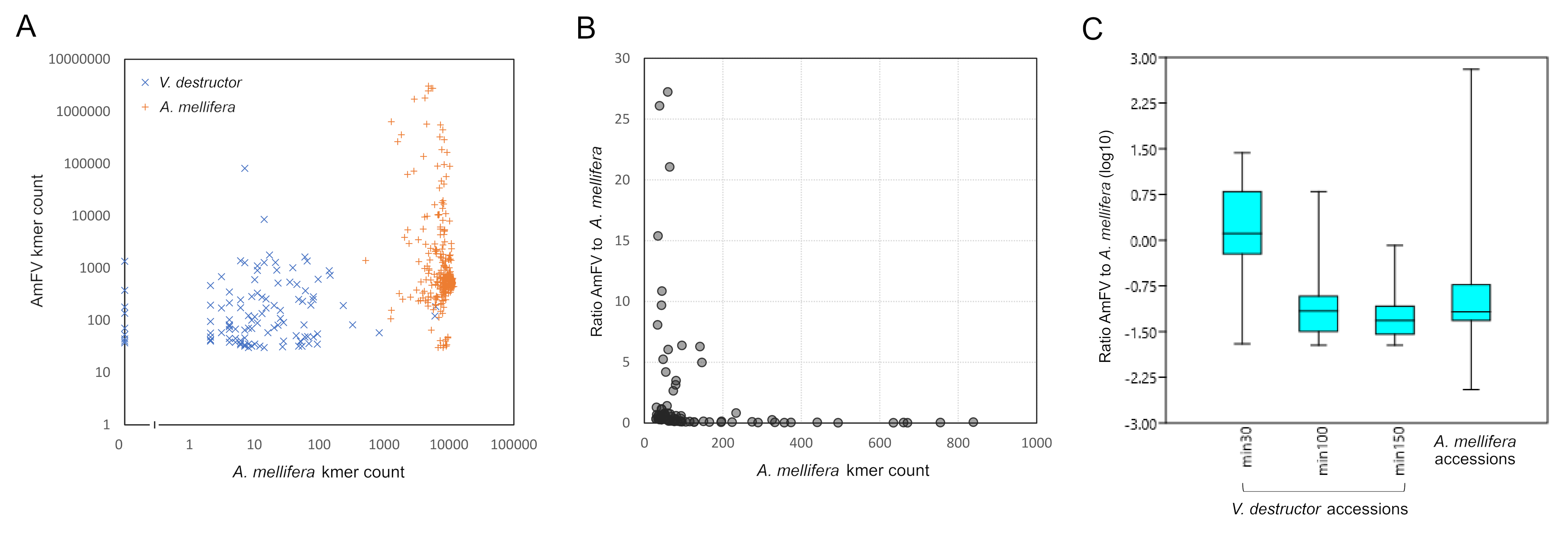

Supplement: Supplemental Information 9 — A. Points represent total AmFV kmer counts for AmFV-positive accessions inV. destructorand A. mellifera, as a function of totalApis melliferakmer counts in those accessions. Some Varroa accessions have high AmFV kmer counts in the absence of any detected A. mellifera kmers. The circled point is a strong outlier of AmFV kmer counts discussed in the text. B. High ratios of AmFV kmers to A. melliferakmers were found for someV. destructoraccessions, but only when the total A. mellifera counts were low. C. Imposing minimum thresholds on the number of A. mellifera kmer counts in eachV. destructoraccession indicates that with adequate sampling effort, no evidence is found ofV. destructoraccessions with higher AmFV relative abundances than the hosts that they parasitize. [file peerj-11-16455-s009.png]

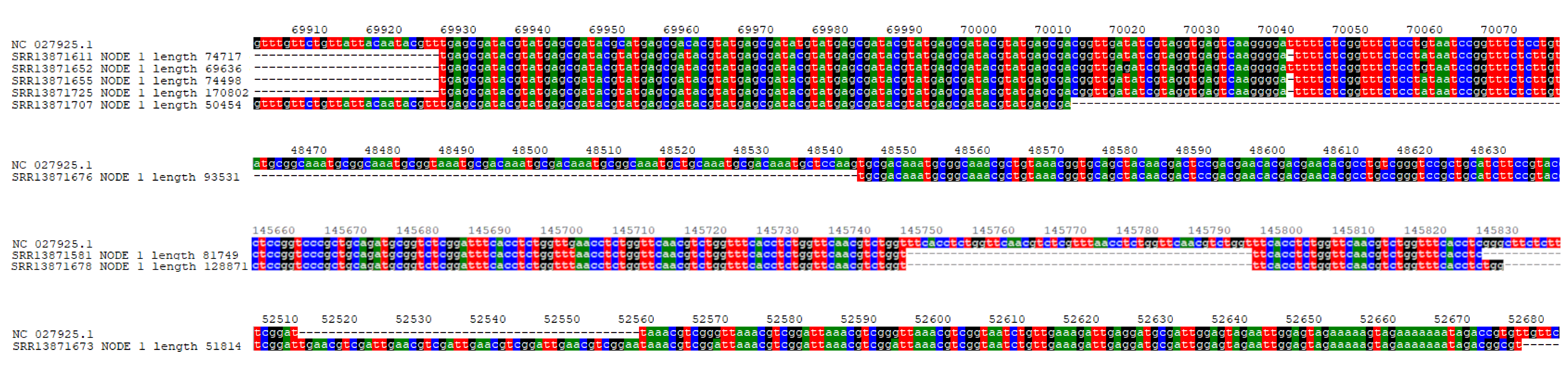

Supplement: Supplemental Information 10 — Four examples of genomic locations in which contigs greater than 50 kb begin or end near a tandem repeat. In each case, one or more contigs are shown aligned to reference genome NC_027925.1. The contig name is that generated by the assembly program, preceded by the accession identifier. [file peerj-11-16455-s010.png]

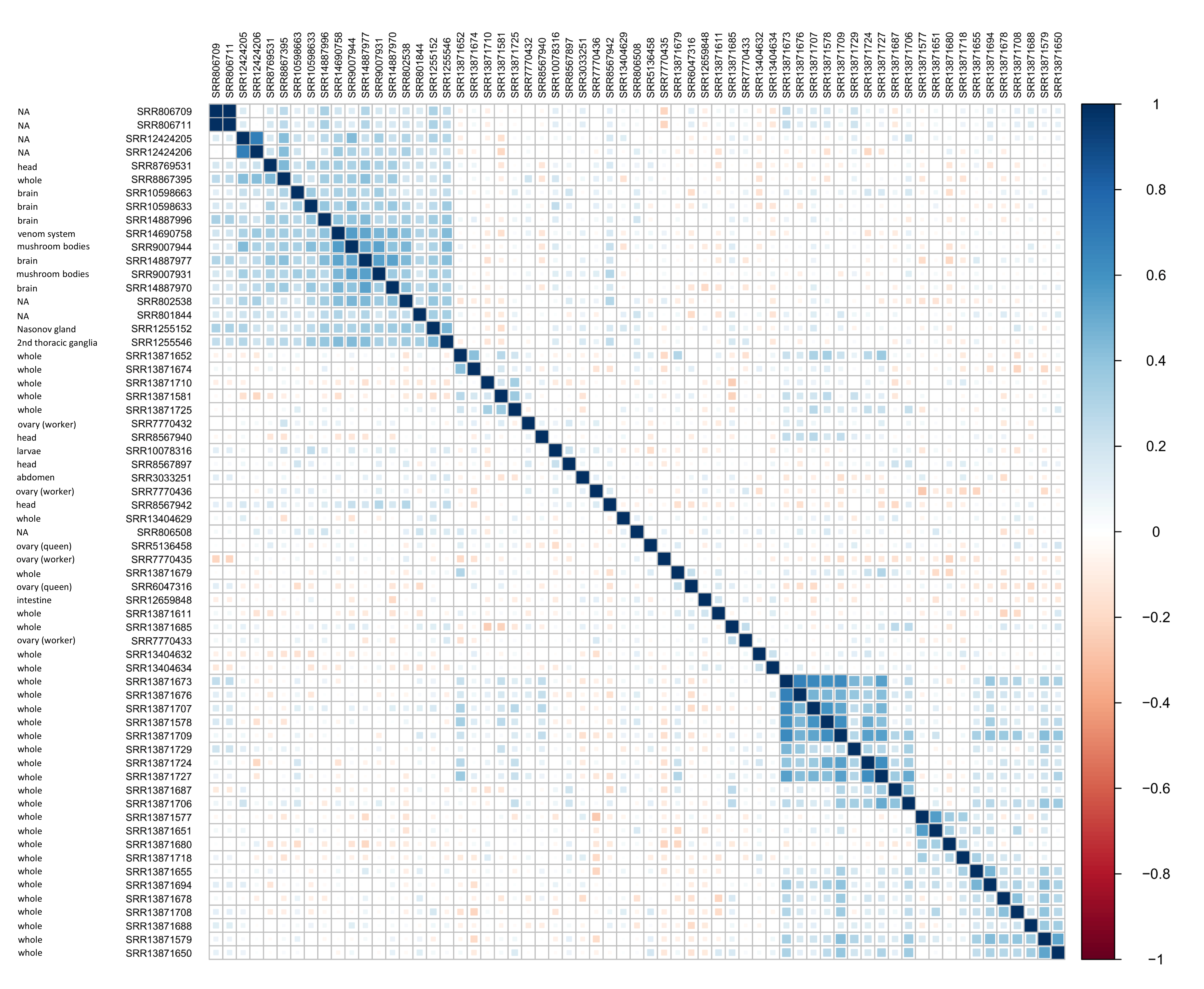

Supplement: Supplemental Information 11 — Each cell of the matrix represents the pairwise Spearman correlation coefficient between 63 RNA accessions with AmFV abundances above minimum thresholds. Accession IDs are listed along with the tissue analyzed, as specified in the associated metadata, not as binned in this study (see text for details). Positive correlations are shaded blue and negative correlations are shaded red, as indicated by the legend. Samples are sorted by hierarchical clustering using Ward’s method. The underlying expression values for ORFs in samples were transformed using a log-ratio approach (see text for details). [file peerj-11-16455-s011.png]
